# Lipidomic profiling of influenza A virus production in MDCK cells towards targeted clone selection

**DOI:** 10.1038/s41598-025-33499-1

**Published:** 2026-01-07

**Authors:** Jocelyn A. Menard, Tilia Zinnecker, Elena Godbout, Joshua A. Roberts, Rozanne Arulanandam, Andrew Chen, Anne Landry, Christopher N. Boddy, Udo Reichl, Jean-Simon Diallo, Yvonne Genzel, Jeffrey C. Smith

**Affiliations:** 1 Department of Chemistry, Carleton University, Ottawa, ON Canada; 2 Max Planck Institute for Dynamics of Complex Technical Systems, Magdeburg, Germany; 3 Centre for Cancer Therapeutics, Ottawa Hospital Research Institute, Ottawa, ON Canada; 4 Department of Chemistry and Biomolecular Sciences, University of Ottawa, Ottawa, ON Canada; 5 Chair of Bioprocess Engineering, Otto von Guericke University, Magdeburg, Germany; 6 Department of Biochemistry, Microbiology and Immunology, University of Ottawa, Ottawa, ON Canada; 7 Institute of Biochemistry, Carleton University, Ottawa, ON Canada; 8 Carleton Mass Spectrometry Centre, Carleton University, Ottawa, ON Canada

**Keywords:** Biochemistry, Biological techniques, Biotechnology, Microbiology

## Abstract

High-yield influenza virus production is essential for efficient vaccine manufacturing to support global demands. Using Madin-Darby canine kidney (MDCK) cells to produce influenza viruses is an attractive alternative to the conventional method of manufacturing vaccines using embryonated eggs. MDCK cells exhibit heterogeneity which can impact viral yields. However, the factors driving the variation between MDCK cells are not fully understood. Utilizing an untargeted liquid chromatography-mass spectrometry lipidomic approach, we investigated two proprietary MDCK clones (C59 and C113) provided by Sartorius (Germany) that differ in biochemical and viral production properties and examined their lipid profiles and dynamics upon influenza A virus (IAV) infection between 24 and 72 h. C113, a high-yield clone, displayed elevated levels across all lipid classes, aside from ether lipids compared to C59, a clone with superior growth properties. IAV infection in clone C59 and C113 displayed key differences, specifically triacylglycerols. Analysis of progeny virions from C59 and C113 clones revealed subtle differences with a positive correlation in lipid profile (*R*^*2*^ = 0.77), suggesting similar lipid raft domains between clones. Overall, these findings highlight specific cellular lipid signatures associated with high-yield production and demonstrate the value of integrating lipidomics methods into biomanufacturing pipelines, providing complimentary quality assurance markers.

**Supplementary Information:**

The online version contains supplementary material available at 10.1038/s41598-025-33499-1.

## Introduction

High-yield influenza virus production has become increasingly important for efficient vaccine manufacturing to support global protection of vulnerable groups and achieve pandemic preparedness. After many decades of solely egg-based production, adherent and suspension Madin-Darby Canine Kidney (MDCK) cells have emerged as a preferred host cell line for influenza virus production due to their susceptibility to a broad range of circulating strains and efficient virus replication. Thus, many MDCK cell lines have been established for vaccine manufacturing by academic and industrial research groups^1^. A key demand in modern vaccine manufacturing is the use of a chemically defined medium that provides the necessary nutrients and allows for suspension cell growth which can drastically improve process scalability. For instance, the seasonal influenza vaccine Optaflu/Flucelvax Tetra (CSL Seqirus) is produced using a proprietary suspension MDCK cell line under serum-free and protein-free conditions^2^. Although the monoclonal origin of a MDCK cell line is not currently a regulatory requirement for vaccine production, selecting the ideal clone is becoming increasingly important as MDCK cells, like many other continuous cell populations used in manufacturing, are well known to exhibit a high degree of cell-to-cell heterogeneity that could be positively exploited. This heterogeneity yields significant differences in morphology, electrophysiology, biochemistry, carbohydrate representation, and in virus production capacities^3–5^. Studies at the single-cell level have detected approximately 1,000-fold differences in progeny virus yields and intracellular viral RNA levels^6^. Although the main causes of this heterogeneity and the factors involved in high-yield production have not yet been clearly identified, high-yield clones are attractive targets for cell line development^7^.

The influenza A virus (IAV) life cycle starts with entering the host cell, followed by genome replication and protein production. After assembly, the virus acquires its envelope by budding from the plasma membrane of the host cell in areas abundant in lipid rafts that are rich in cholesterol and sphingolipids (SLs)^8,9^. This results in a viral envelope enriched in these lipids relative to the lipid profile of the host cell^8,10,11^. IAV relies on host cell metabolic systems, including lipid metabolism, to complete its life cycle. At the same time, host cells activate a variety of defense mechanisms to restrict viral replication and maintain cellular integrity. These include the regulation of metabolic pathways, activation of signaling cascades, induction of apoptosis, and cytopathic responses such as cell lysis^12,13^. These interactions lead to concerted biomolecular dynamics in each stage of infection to maintain cellular integrity, energy balance, responsive signaling mechanisms and support viral packaging. All of these factors affect viral yield^12,14^. Moreover, host cell lines with different IAV sensitivities have been shown to exhibit different diacyl, lysolipid, and SL metabolism regulation, offering insights into pathogenicity mechanisms of IAV infection^15^. The high degree of heterogeneity among different MDCK sublines has been documented, and variation in glycosphingolipid (GSL) composition has been reported^16–18^, however, a comprehensive investigation of lipid composition differences in MDCK cells has not been conducted. Thus, investigating the cellular lipid composition and metabolism of MDCK sublines presents an opportunity to better understand the biomolecular mechanisms of intracellular viral replication and also guide the selection of host cell clones with optimized lipid profiles to improve IAV production.

Driven by the development of novel technologies, mass spectrometry (MS)-based techniques have become the gold standard to study lipids^19^. Untargeted lipidomics workflows can identify and elucidate structural data on hundreds of lipids from very small quantities of biological material, providing new insights into their biological functions^20^. These developments have enabled wider applications of lipidomics, including biotherapeutics manufacturing^21,22^. For instance, we recently applied an untargeted lipidomics workflow to third-generation lentiviral vectors produced in HEK 293T packaging cells. Our study detected over 150 lipid species in both cells and viral particles, identified more than 80 lipids with significant changes during virus production, and distinguished over 100 virion-specific lipids in the concentrated vector product, demonstrating how lipidomics profiling can inform quality control and guide optimization of vector composition for enhanced transduction efficiency[2121].

This study details comprehensive comparative lipidomics analyses of two proprietary monoclonal suspension MDCK cell lines provided by Sartorius (Germany), originating from a joint cell population, with both lines being candidates for influenza vaccine manufacturing. Termed C59 and C113, these cell lines differ from each other in size, cell growth kinetics, nutrient consumption rates, and virus production, while being cultured in the same medium and under the same process conditions. Applying the liquid chromatography (LC)-MS-based untargeted lipidomics pipeline that we previously established for lentivirus production in HEK 293T cells^21^, we have elucidated and compared the lipid profiles of each MDCK cell clone at 24, 48, and 72 h post infection (hpi) with the aim to identify specific molecular signatures associated with high viral yields. Moreover, we compared the lipid profiles of purified virus produced from each clone to investigate how the of the host cell impacts the composition of the virus membrane. This approach offers the potential to improve the characterization of virus-host cell interactions and the impact of host cell selection on virus yield. In the future, it will help to optimize current IAV production systems, and advance viral vaccine manufacturing.

## Results

**Fig. 1:**
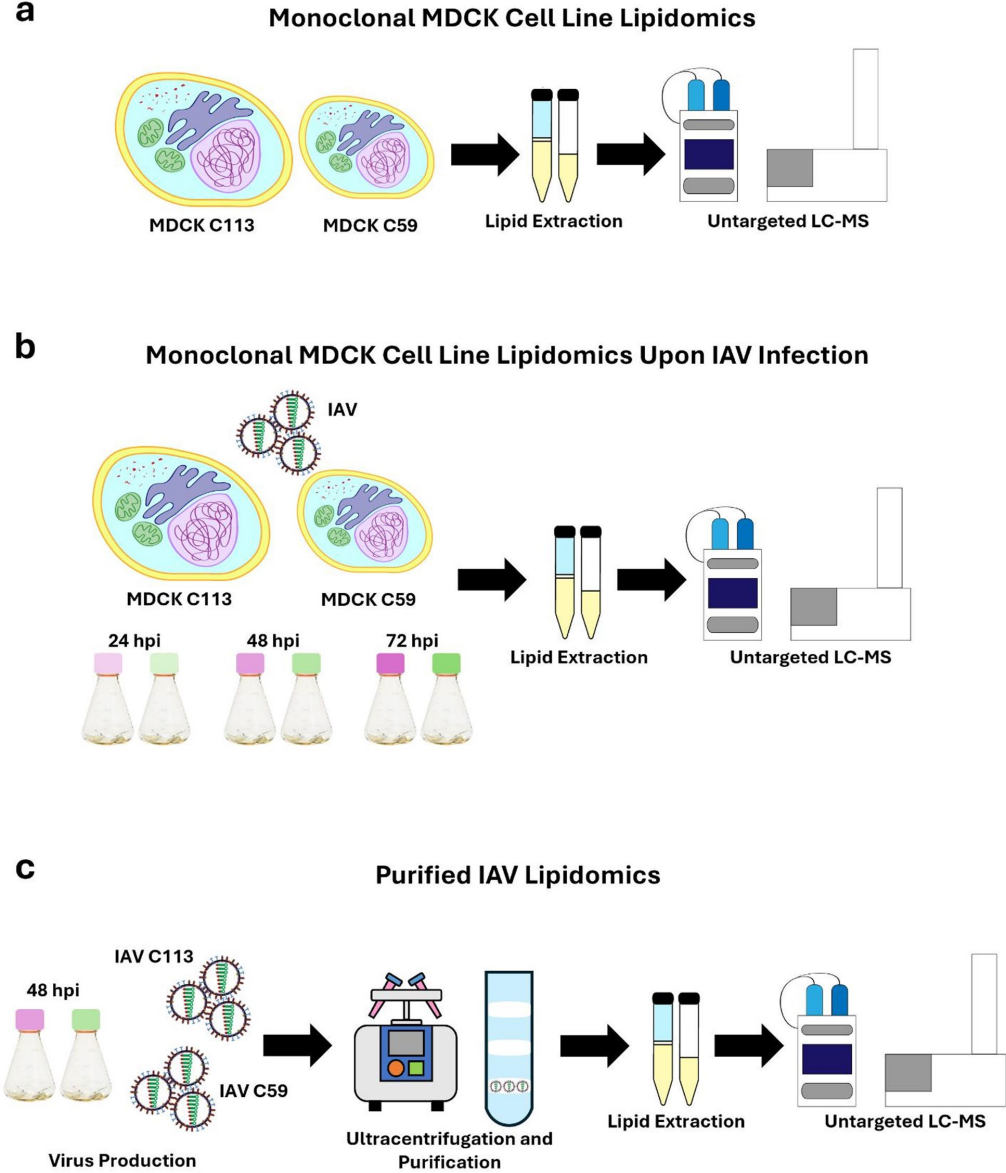
Lipidomics workflow to investigate IAV production in two clonal MDCK cell lines. (**a**) Analysis of uninfected MDCK monoclonal cell lines (control), C59 and C113, at 72 h. (**b**) Analysis of control and IAV infected MDCK cells at 24, 48, and 72 hpi. (**c**) Analysis of purified IAV from the supernatant of each clone harvested at 48 hpi.

The lipid profiles of C59 and C113 were analyzed first (Fig. 1A) as it is known that they differ in cell diameter, growth kinetics, nutrient consumption, and virus production^7^. C59 and C113 cells were then seeded at 2 × 10^6^ cells/mL in shake flasks and infected with IAV (A/PR/8/34, H1N1) in duplicates. Cultivations were monitored regarding cell growth (viable cell concentration (VCC), diameter, metabolite concentration) and IAV production analyses (infectious and total virus titer) at 24 h intervals; uninfected cells served as controls (Fig. 1B). For lipid analysis, lipids were extracted at each sample point from a total of 2 × 10^6^ cells and analyzed via LC-MS. Finally, we analyzed the lipid profile of IAV particles purified from C59 (IAV C59) and C113 (IAV C113) from sample time point 48 hpi (Fig. 1C).

### MDCK C59 and C113 cell clones have distinct lipid profiles

**Fig. 2:**
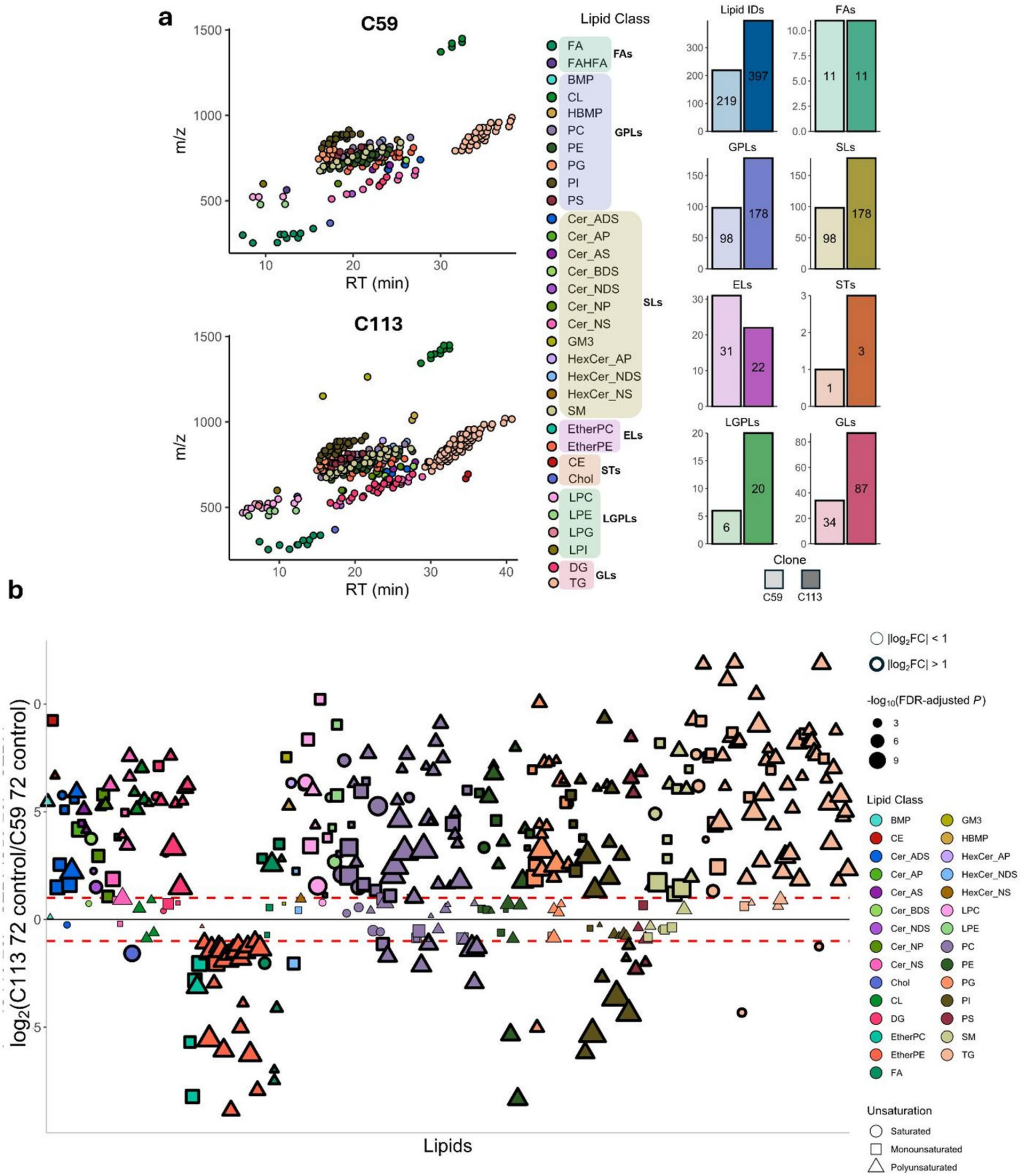
MDCK C59 and C113 cell clones have distinct lipid profiles. (**a**) Scatterplots representing the identified lipids in both clones based on their retention time (RT) and *m/z* ratio. The number of identified lipids within sub-classes between each clone is represented in the figures on the right with y-axis showing number of lipids. (**b**) Bubble plot of C113 compared to C59 including only significant fold changes (false discovery rate (FDR)-adjusted *p* < 0.05) (*n* = 366). All *p* values were calculated using Student’s t-test (2-tailed, unpaired with unequal variance) with FDR-correction (*n* = 6, two biological replicates with three technical replicates). Positive values indicate a greater abundance for C113, negative values indicate a greater abundance for C59. Lipids on x-axis are sorted based on alphabetical order. The dashed red lines represent the log_2_FC ± 1. Lipids below and above these lines are bolded. The size of the symbols is proportional to the -log_10_(FDR-adjusted *p* value). The shape of the symbols represents the number of unsaturation(s) on the lipid.

Uninfected C59 and C113 cells were cultured for 72 h and analyzed via an untargeted lipidomics analysis to characterize and contrast both cell lines. Cells were seeded at 2 × 10^6^ cells/mL and reached maximum VCCs of 9.04 × 10^6^ cells/mL (C59) and 4.42 × 10^6^ cells/mL (C113), as illustrated in Supplementary Fig. S1. Lipids extracted from the collected cell pellets were subjected to LC-MS analysis that revealed distinct lipid profiles between the two cell lines (Fig. 2). A total of 438 lipid species were detected in both cell lines, 136 in common. 219 lipid species were identified in C59 (Supplementary Table S1, 103 and 116 in positive and negative ion modes, respectively), and 397 lipid species were identified in C113 (Supplementary Table S2, 218 and 179 in positive and negative ion modes, respectively), as illustrated in Fig. 2A. C113 yielded increased numbers of glycerophospholipids (GPLs), SLs, sterols (STs), lysoglycerophospholipids (LGPLs), and glycerolipids (GLs), whereas C59 yielded a greater number of ether lipids (ELs). An equal number of free fatty acids (FAs) were identified in both clones. Comparing the relative intensities of the major lipid classes between each clone indicated that C59 is enriched in cholesterol, ether-linked phosphatidylcholine (EtherPC), ether-linked phosphatidylethanolamine (EtherPE), FA, phosphatidylcholine (PC), phosphatidylethanolamine (PE), and phosphatidylinositol (PI) species, while C113 is enriched in cholesteryl ester (CE), ceramide (Cer), cardiolipin (CL), diacylglycerol (DG), hexosylceramide (HexCer), LGPL, phosphatidylglycerol (PG), and triacylglycerol (TG) species (Supplementary Fig. S2). The largest distinction between the clones was observed in TG species with a relative intensity difference of 19.6%. Since TG and CE species are predominantly found in LDs^23^, the analysis was repeated after removing these species from the dataset to contrast the membrane lipid compositions of the two cell lines (Supplementary Fig. S2). No significant changes were observed for PC and PE species (Supplementary Fig. S2), which are the main lipid components of MDCK cell membranes^8^. C113 had higher relative intensities of Cer, DG, LGPL, PG, and sphingomyelin (SM) species, while C59 had higher relative intensities of cholesterol, EL, and PI species (Supplementary Fig. S2).

Out of the 438 lipids identified, 366 were determined to have a statistically significant fold change (FC) (FDR-adjusted *p* < 0.05) between the two clones (Fig. 2B and Supplementary Table S3). Of the 366 significant lipids, 237 had a log_2_FC > 1 whereas 58 lipids had a log_2_FC < -1 (Supplementary Table S3), the remainder had a significant log_2_FC between ± 1. A list of the top 10 most increased and decreased lipid species is also included in Supplementary Table S3. Overall, C113 had increased lipid levels across several lipid classes compared to C59. Lipid classes that were depleted in C113 compared to C59 included cholesterol, EtherPC and EtherPE. TG species were observed to be in greater abundance in C113 with the exception of two saturated species, TG 48:0 and TG 16:0_16:0_18:0. PE species were also relatively higher in C113, with the exception of PE 18:0_20:3 and PE 18:1_20:3. PI species had varied levels in C113 compared to C59, with 17 having a log_2_FC > 0 and 13 with a log_2_FC < 0 including three PI species with a 20:3 fatty acid. The only PG species greater in C59 with a log_2_FC < -1 also contained a 20:3 fatty acid (PG 16:0_20:3). PC 18:1_20:3 and PC 16:1_20:3 were amongst the most increased in C113 (log_2_FC = 9.1 and 7.7, respectively). FA species had varied levels within both clones; however, the most unsaturated FAs (FA 20:4 and FA 20:6) were depleted in C113 relative to C59.

**Fig. 3:**
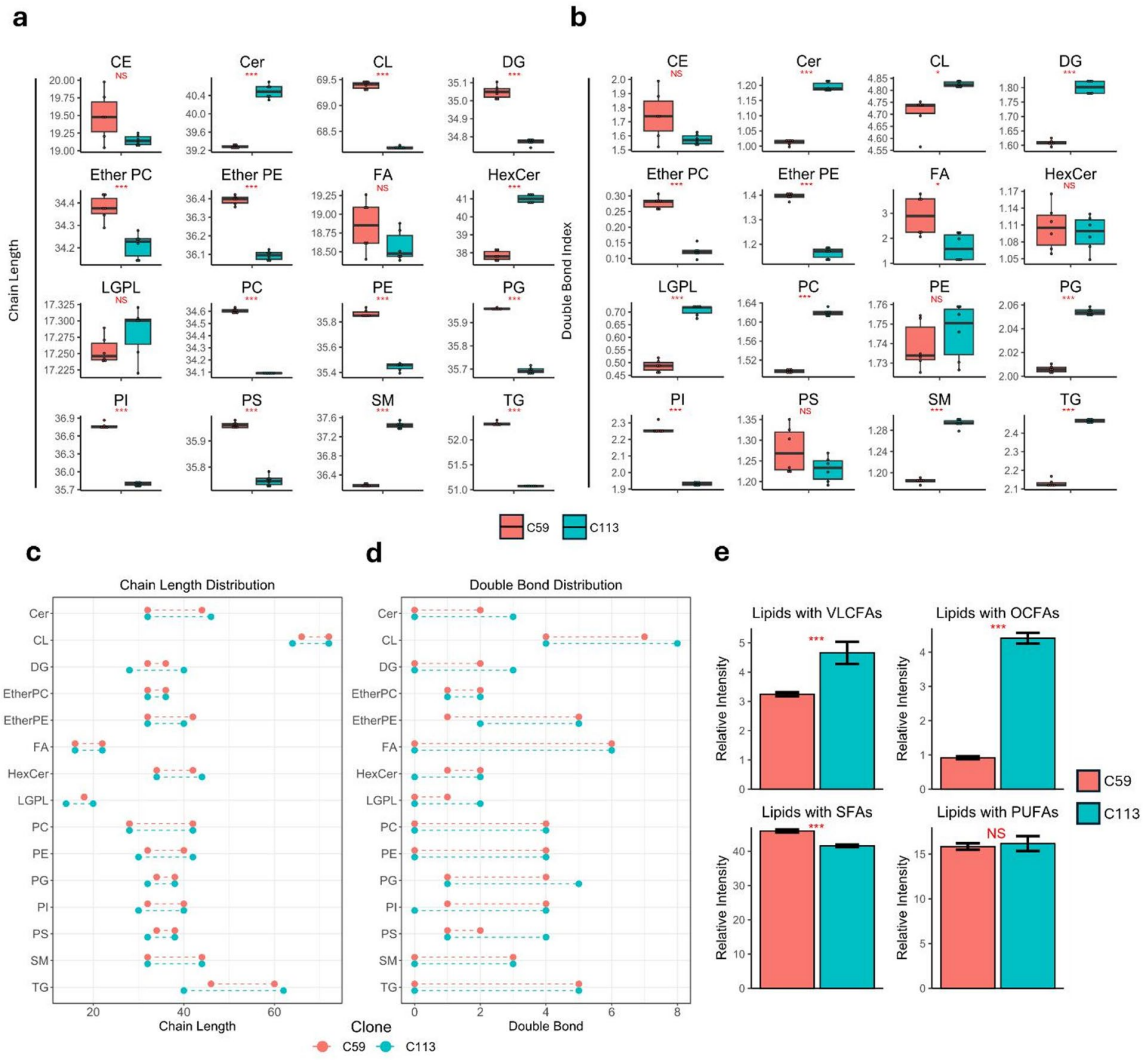
MDCK C59 and C113 cell clones differ in total chain length and double bond index. Box plots representing (**a**) the weighted average total chain length and (**b**) the double bond index between clones for each lipid class. Line plots demonstrating the range of (**c**) total chain length and (**d**) double bonds between clones for each lipid class. (**e**) Bar plots demonstrating the relative intensities of lipids with very-long chain fatty acids (VLCFAs), odd-chain fatty acids (OCFAs), saturated fatty acids (SFAs), and polyunsaturated fatty acids (PUFAs) between the clones. All *p* values were calculated using Student’s t-test (2-tailed, unpaired with unequal variance) with FDR-correction (*n* = 6, two biological replicates with three technical replicates). Shown in red under the figure titles: NS, *p* > 0.05, *, *p* ≤ 0.05, **, *p* ≤ 0.01, ***, *p* ≤ 0.001. Error bars indicate the standard deviation between replicates (*n* = 6).

MDCK clones C59 and C113 also differed in their carbon chain lengths and double bond indices of their FAs within each lipid class (Fig. 3). For ceramide-containing lipids (Cer, HexCer, SM), C113 had a longer weighted average chain length compared to C59, while GPLs (CL, EtherPC, EtherPE, PC, PE, PG, PI, phosphatidylserine (PS)) and GLs (DG, TG) were observed to have significantly shorter chain lengths (Fig. 3A). C59 was observed to have a lower double bond index across Cer, CL, DG, LGPLs, PC, PG, SM and TG species, and a higher index for EtherPC, EtherPE, and PI species (Fig. 3B). C113 also demonstrated a wider distribution of chain length and number of double bonds within identified lipid species, with the exception of EtherPEs (Fig. 3C and D). The relative intensity of lipids containing very-long chain fatty acids (VLCFAs), odd-chain fatty acids (OCFAs), and saturated fatty acids (SFAs) also differed between C113 and C59 (Fig. 3E). C113 had elevated levels of lipids with VLCFAs and OCFAs, while C59 had higher levels of lipids with SFAs. No significant differences were observed between lipids with polyunsaturated fatty acids (PUFAs). Overall, untargeted lipidomics analysis revealed distinct lipid profiles between the two monoclonal cell lines, with ELs and TG species showing the most pronounced differences. The relative abundances of the most common membrane structural lipids, PC and PE, were not observed to be statistically different between the cell lines.

### IAV infection leads to unique lipid dynamics between MDCK C59 and C113 cell clones

**Fig. 4:**
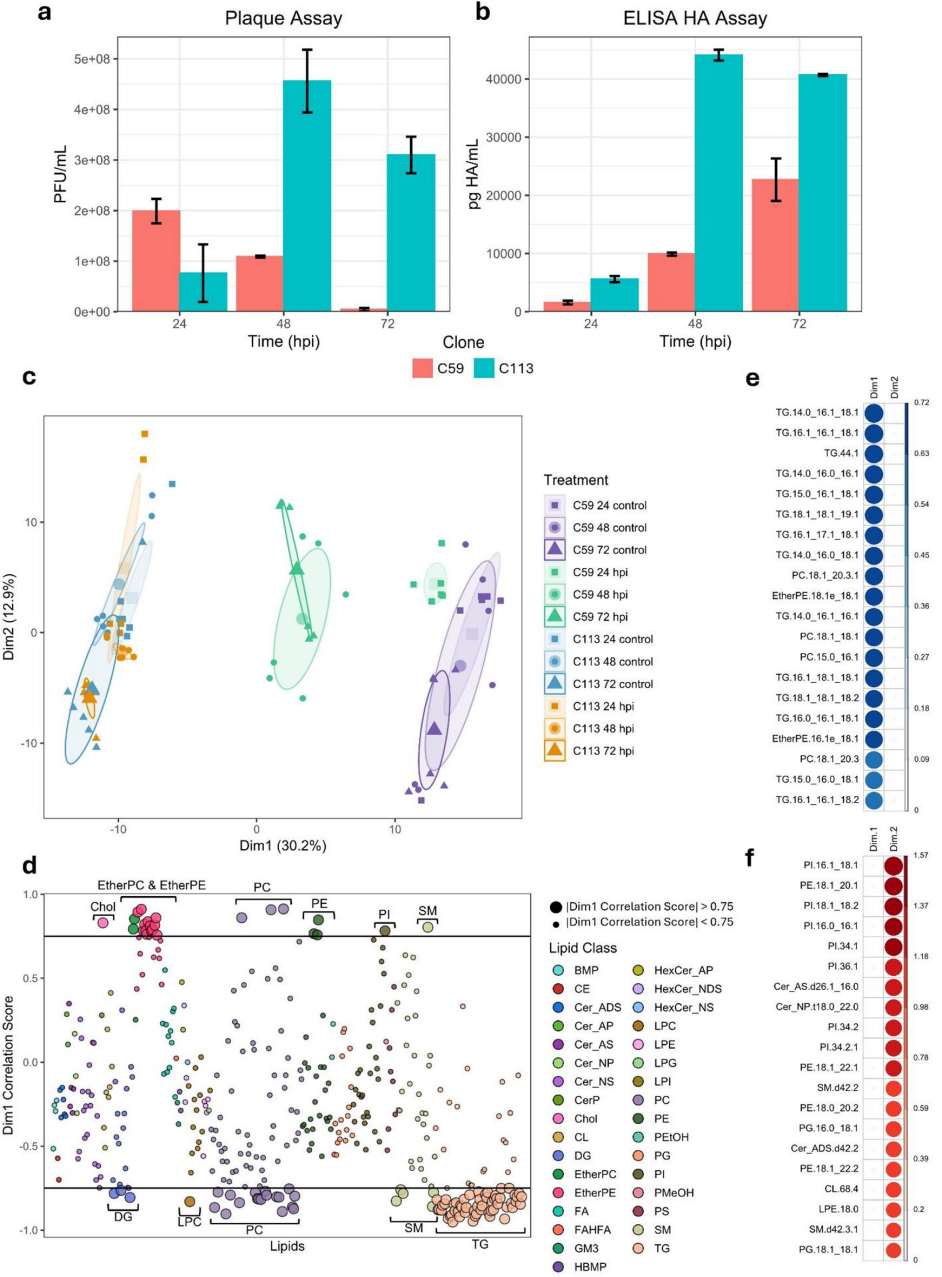
Analysis of the lipid dynamics of IAV-infected MDCK C59 and C113 cell clones. IAV quantification using (**a**) plaque assay and (**b**) hemagglutinin (HA) ELISA. Errors bars represent the standard deviation between replicates (*n* = 2). (**c**) Principal component analysis (PCA) of all MDCK samples, including C59 and C113 control and infected samples at 24, 48, and 72 hpi. (**d**) Plot of lipids and how they contribute to the variance observed in Dim1. The lipids contributing most to Dim1 (|correlation score| > 0.75) are represented by larger points. The 20 lipids with the highest contribution to the difference observed in (**e**) Dim1 and (**f**) Dim2. TG and EL species drove the large variance observed in Dim1 whereas the smaller variance observed in Dim2 is explained by a variety of lipids.

**Fig. 5:**
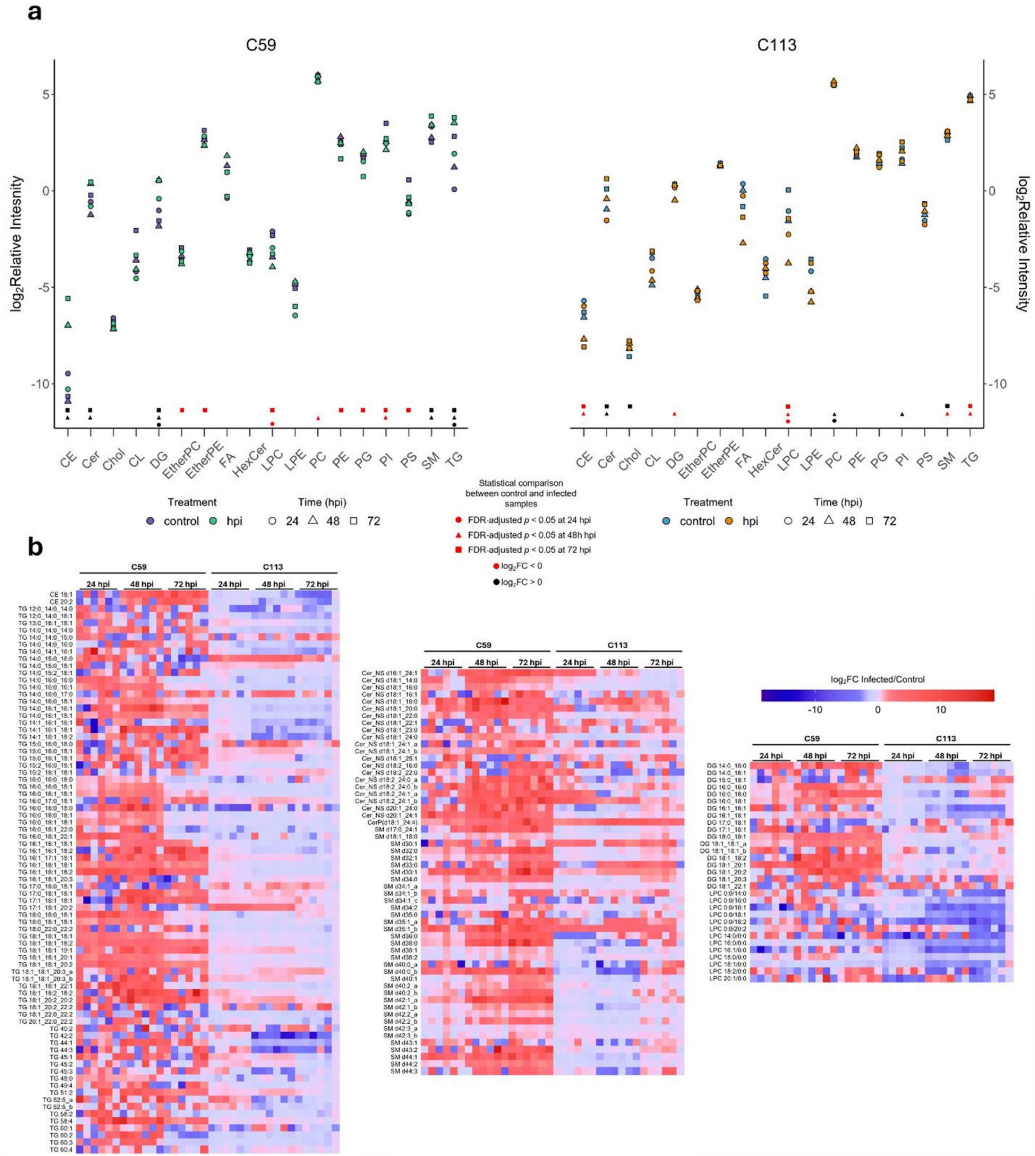
A statistical comparison reveals that CE, TG, SL, DG and LPC species are the most impacted in IAV-infected MDCK C59 and C113 cells clones. (**a**) The log_2_ relative intensity of lipid classes in control and infected samples from C59 and C113 at each timepoint. Statistical comparison between control and infected samples are represented above the x-axis with each shape representing a timepoint and the color representing a positive or negative log_2_FC. (**b**) Complementary heatmaps of CE, Cer_NS, CerP, DG, LPC, SM, and TG species representing the log_2_FC between infected and control samples for C59 and C113 at each timepoint. All *p* values were calculated using Student’s t-test (2-tailed, unpaired with unequal variance) with FDR-correction (*n* = 6, two biological replicates with three technical replicates). Each square represents a log_2_FC for a total of 6 squares per sample (2 biological replicates with 3 technical replicates.

To analyze the effect of IAV infection on the lipidome of C59 and C113 cells, experiments were repeated following viral infection. IAV infected C59 and C113 cells, as well as uninfected controls, were cultured for 24, 48 and 72 hpi and the VCC, cell size (Supplementary Fig. S1), virus production (Fig. 4A and B), and lipid composition (Figs. 4 and 5) were measured for each cell type at each time point. At all timepoints, C59 yielded a smaller diameter (Supplementary Fig. S1) and lower glucose consumption rate compared to C113 (Supplementary Fig. S3). Following lipid analysis, a principal component analysis (PCA) of all samples showed clear separation in dimension 1 (Dim1) between C59 and C113 across controls and infected samples (Fig. 4C and Supplementary Table S4), representing 30.2% of the total variance. The lipids that contributed the most to Dim1 separation were Chol, DG, EtherPC, EtherPE, LPC, PC, PE, PI, SM, and TG species (Fig. 4D and E). Minimal separation was observed between groups in dimension 2 (Dim2), which represented 12.9% of the total variance, with the top contributing variables in Dim2 being GPLs (PI, PE, PG), suggesting these classes contribute relatively less to the differences observed in infection dynamics between C59 and C113 (Fig. 4C and F).

C59 achieved its maximum infectious viral titer at 24 hpi, while C113 had an approximately 3-fold higher maximum infectious viral titer at 48 hpi (Fig. 4A). 24 hpi C59 control and infected samples were separable via PCA, while C113 control and infected samples clustered together (Fig. 4C) indicating minimal change in lipid dynamics in C113 cells and highlighting lipid species exhibiting the most pronounced infection-related changes in C59 cells. C113 achieved its peak infectious titer (Fig. 4A) and total titer (Fig. 4B) at 48 hpi, with control and infected samples clustering together indicating minimal lipid composition changes (Fig. 4C). PCA displayed separation between C59 control and infected samples at 48 hpi demonstrating that more significant lipid changes occurred once the total titer began to increase significantly (Fig. 4B and C). At 72 hpi, C113 had a higher infectious and total titer compared to C59 (Fig. 4A and B). PCA yielded clustering of C113 control and infected samples at 72 hpi whereas C59 72 hpi infected samples separated from the control samples and clustered with the C59 48 hpi infected samples, reiterating that larger lipid changes occurred once the total titer amounts were higher (Fig. 4B and C). DG and TG species increased in C59, whereas LPC levels decreased in both C59 and C113 (Fig. 5A and B). PC species increased at 24 hpi in C113 (Fig. 5A). CE, ceramide-1-phopshate (CerP), ceramide non-hydroxy-sphingosine (Cer_NS), DG, SM, and TG species increased in C59 after infection, while C113 yielded increased levels of Cer (CerP and Cer_NS), PC, and PI species (Fig. 5A, B and Supplementary Fig. S4). Depletion of PC and SM species was observed in C59, whereas C113 decreased in CE, LPC, SM, and TG species (Fig. 5A and B). Widespread depletion of GPLs (EtherPC, EtherPE, PE, PG, PI, and PS) was observed in C59 with an increase in CE, Cer (CerP and Cer_NS), DG, SM, and TG species (Fig. 5A, B and Supplementary Fig. S4). Cer (CerP and Cer_NS), cholesterol, and SM species increased in C113 while CE, LPC, and TG species decreased (Fig. 5A, B and Supplementary Fig. S4). Overall, IAV infection impacted the lipid dynamics in C59 and C113 differently. IAV infection in C59 predominantly affected CE, Cer, DG, LPC, SM, and TG species at 48 and 72 hpi, correlating with peak viral production whereas the changes observed in C113 were minor as PCA did not separate the control and infected samples, inferring minimal lipid alterations at any of the timepoints.

### Lipid analysis of purified IAV reveals lipid profiles with subtle differences when produced in either C59 or C113 cell clones

**Fig. 6:**
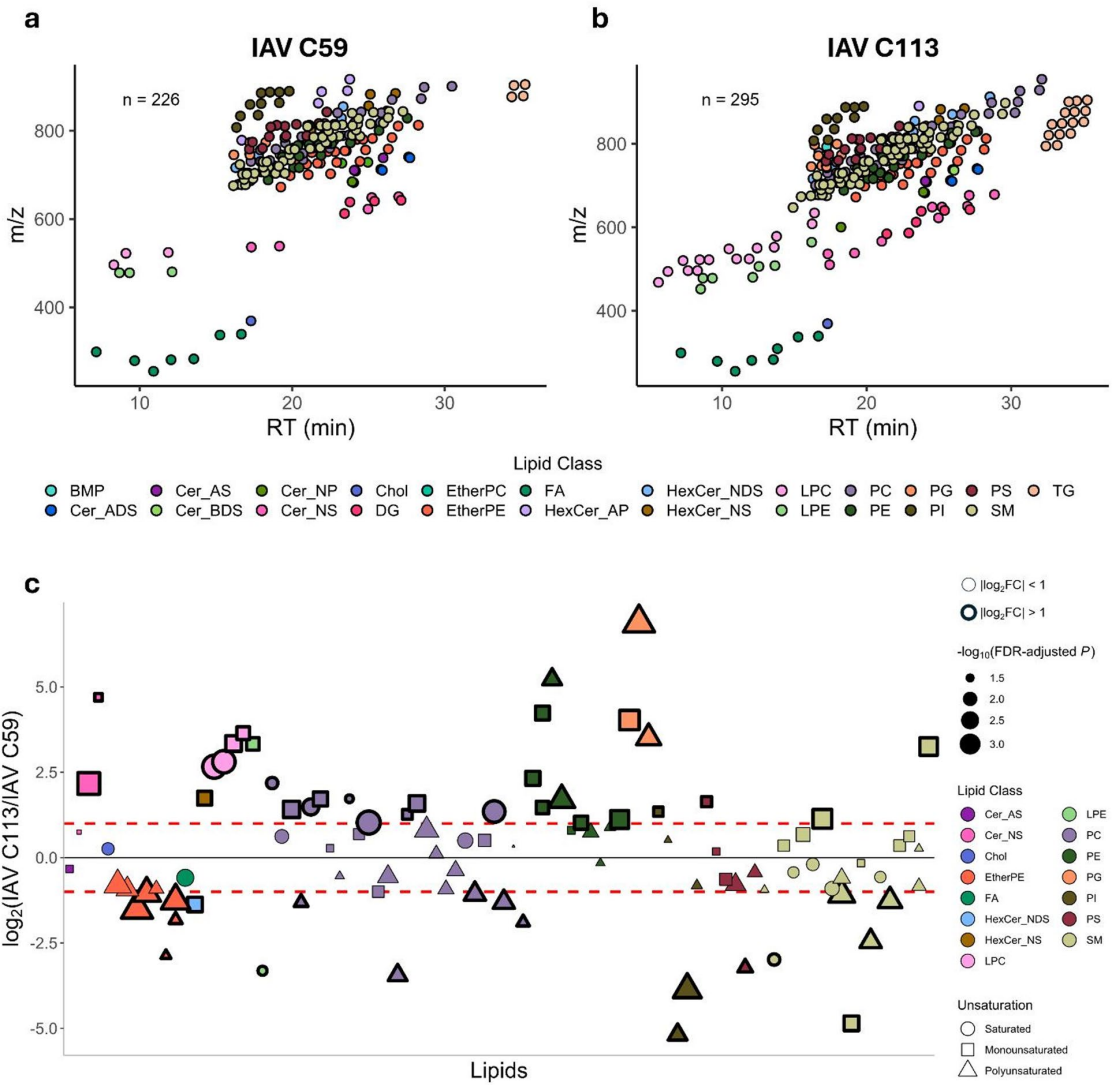
Analysis of purified IAV lipids produced from C59 (IAV C59) and C113 (IAV C113) cell clones. Scatterplot representing the lipids identified based on their retention time and *m/z* ratio in (**a**) IAV C59 and (**b**) IAV C113. (**c**) Bubble plot of IAV C113 compared to IAV C59 including only significant fold changes (FDR-adjusted *p* < 0.05) (*n* = 95). All *p* values were calculated using Student’s t-test (2-tailed, unpaired with unequal variance) with FDR-correction (*n* = 3, one biological replicates with three technical replicates). Positive values indicate a greater abundance for IAV C113, negative values indicate a greater abundance for IAV C59. Lipids on x-axis are sorted based on alphabetical order. The dashed red lines represent the log_2_FC ± 1. Lipids below and above these lines are bolded. The size of the symbols is proportional to the -log_10_(FDR-adjusted *p* value). The shape of the symbols represents the number of unsaturation(s) on the lipid.

To investigate the impact of production cells on the viral particle lipidome, IAV was purified from C59 and C113 cells at 48 hpi and independently analyzed. Virus quantification was performed following gradient purification with C59 achieving 5.5 × 10^6^ PFU/mL and C113 obtaining 1.7 × 10^10^ PFU/mL. Lipid analysis of IAV C59 yielded 226 lipids from 22 different classes (Fig. 6A and Supplementary Table S5) and analysis of IAV C113 identified 295 lipids spanning 23 lipid classes (Fig. 6B and Supplementary Table S6); a total of 306 unique lipids were identified between both samples. The same lipid classes were present in both samples with the exception of bis(monoacylglycerol)phosphate (BMP), which was only identified in IAV C113. Of the 306 lipids, 95 had significant FCs comparing IAV C113 to IAV C59 and of these, 31 lipids had a higher relative abundance in IAV C113 (log_2_FC > 1) and 20 lipids had a higher relative abundance in IAV C59 (log_2_FC < -1), (Fig. 6C and Supplementary Table S7). A list of the top 10 most increased and decreased lipid species is also included in Supplementary Table S7. LPC, LPE, PE, and PG species were more abundant in IAV C113 while IAV C59 was enriched in EtherPE species. PC species had varying levels across both viral products with a general trend of saturated and monounsaturated species being more abundant in IAV C113 while polyunsaturated species were more abundant in IAV C59. PC species with OCFAs (PC 16:0_17:0, PC 15:0_16:0, and PC 15:0_20:1) were all increased in IAV C113. Polyunsaturated PI species (PI 18:1_20:3 and PI 18:0_20:3) were elevated in IAV C59 while monounsaturated PI 16:0_16:1 was the only PI species increased in IAV C113. Two HexCer species were identified with the sphingosine species (HexCer_NS) more abundant in IAV C113 and the dihydrosphingosine species (HexCer_NDS) more abundant in IAV C59. Very long chain Cer_NS species (d20:1_24:0 and d18:1_24:0) were elevated in IAV C113 (log_2_FC > 2) while the shorter Cer_NS d18:1_16:0 species was similar between both populations.

**Fig. 7:**
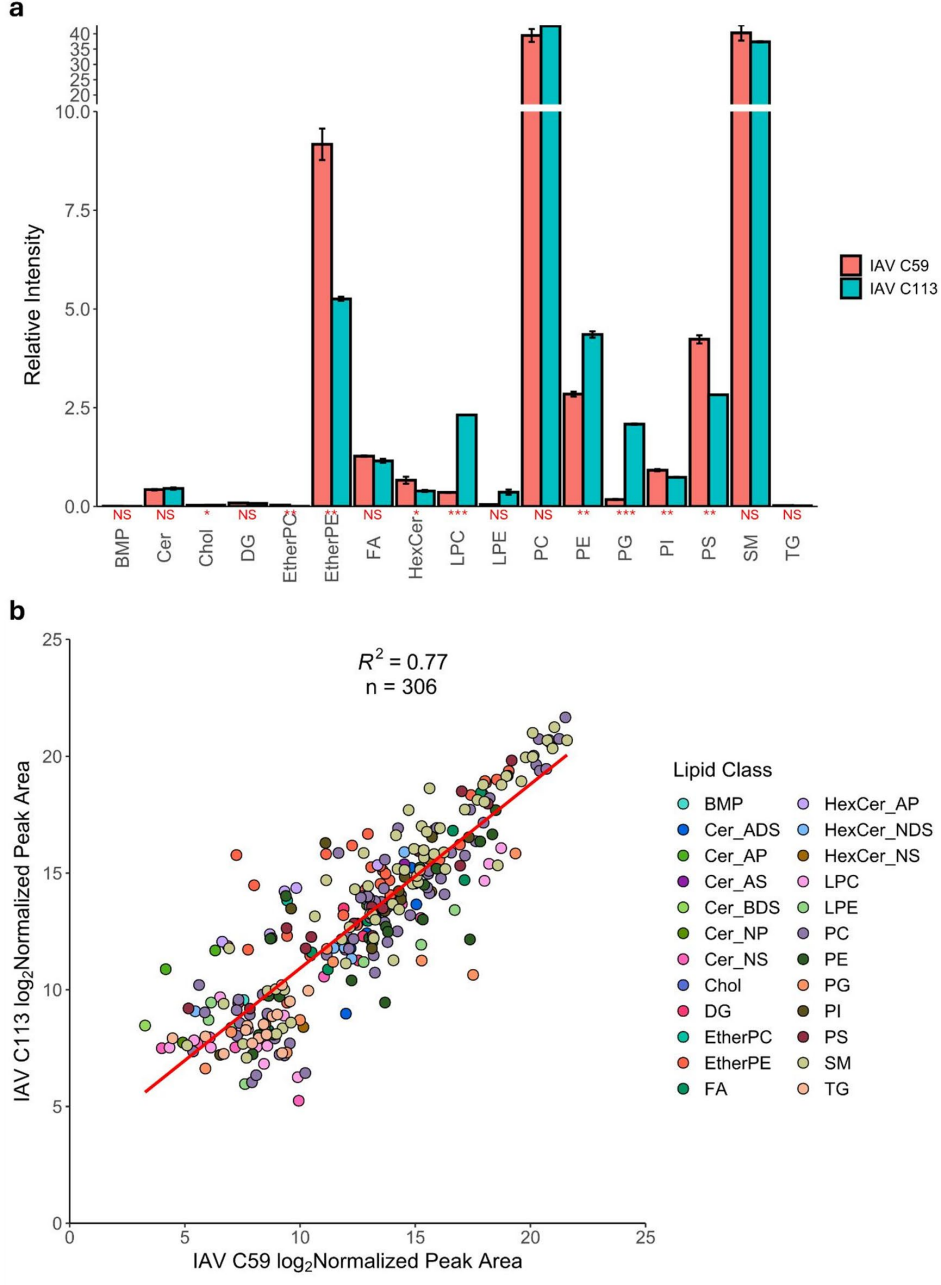
Relative quantification of lipids from purified IAV C59 and IAV C113 yielding similar lipid profiles. (**a**) Bar plot demonstrating the relative intensity of each lipid class between IAV C59 and IAV C113. Shown in red: NS, *p* > 0.05, *, *p* ≤ 0.05, **, *p* ≤ 0.01, ***, *p* ≤ 0.001. Error bars indicate the standard deviation between replicates. (**b**) Scatterplot representing the log_2_ normalized peak area of IAV C59 and IAV C113. All *p* values were calculated using Student’s t-test (2-tailed, unpaired with unequal variance) with FDR-correction (*n* = 3, one biological replicates with three technical replicates). Red line represents the linear fit of the data points.

Despite differences between the lipid envelope of IAV C59 and IAV C113, overarching similarities were observed (Fig. 7). SM and PC levels had no significant difference as far as their relative fraction within the IAV lipid envelope (Fig. 7A). Significant differences in relative intensities included cholesterol, EtherPE, LPC, PE, PG, PI and PS species. Through comparing the intensity of GSL, SM, cholesterol, and Cer species to the intensity of PC species, the enrichment of lipids commonly found in lipid rafts was evaluated (Supplementary Fig. S5). Both IAV C59 and IAV C113 were demonstrated to be enriched in cholesterol, GSL, and SM species and depleted in Cer relative to the host cell (Supplementary Fig. S5). Fatty acid chain analysis demonstrated that IAV C59 had longer GPLs compared to IAV C113, which correlates to the observations made for uninfected C59 and C113 cells (Supplementary Fig. S6 and Fig. 3A). The phospholipids in both IAV C59 and IAV C113 had a lower DBIs compared to their respective host cells (Supplementary Fig. S6 and Fig. 3B) indicating that viral lipidomes are more saturated compared to their production hosts. A Pearson correlation analysis of the log_2_ normalized peak areas of the 306 identified lipids in IAV C59 and IAV C113 yielded a strong positive correlation (*R*^2^ = 0.77) (Fig. 7B), signifying that the lipid profiles of IAV C59 and IAV C113 have a great degree of similarity. Additionally, 18 of the 20 most abundant lipids in IAV C59 and IAV C113 were common to both samples (Supplementary Tables S8 and S9), further suggesting that the progeny virions produced from C59 and C113 show a high degree of concordance.

## Discussion

MDCK cells, which are considered hosts for IAV manufacturing, are a highly heterogeneous population with significant differences in growth properties and virus production capacities. Clone picking and selecting suitable candidates is a promising strategy for developing and optimizing the virus production process. Conclusions regarding specific requirements for high-yield production can be drawn based on clones with favorable properties. To identify specific targets for clone selection, cultivations must be performed under suitable cell growth and infection conditions, and an in-depth characterization of specific cell properties must be performed. Since the envelope of IAV is derived from its host cell, lipidomics may be key to identifying factors correlated with high cellular virus yield. To help address this challenge, we investigated the lipid composition differences between two clonal MDCK cell clones, with the aim of identifying insights that could support more efficient cell culture-based virus production. The cell lines used in this work differ in diameter (C59: 14.8 to 16.5 μm; C113: 15.9 to 18.9 μm), resulting in different average volumes and surface areas. C59 grows at faster rates with higher VCCs compared to C113; however, the glucose consumption rate of C59 is half that of C113^7^. Despite C59 propagating the fastest in cell culture, the number of virions/cell is nearly 8-fold higher in C113 compared to C59^7^. The underlying drivers of MDCK heterogeneity remain elusive and likely stem from a multifaceted interplay of various biological factors^24,25^. Non-lipid metabolic signatures of other high producer cell lines have been reported^24,26–28^. Clonal MDCK H1 cells were shown to have particularly high productivity, with elevated glucose uptake, superior tricarboxylic acid cycle flux, moderate amino acid consumption, better regulation of reactive oxygen species (ROS), and sufficient intracellular energy supply for IAV production^28^. MDCK-2B6 cells had increased cellular susceptibility and cell proliferation activity to IAVs, which promoted viral production^26^. MDCK cells had better virus production in scaled-up bioreactor conditions compared to Vero cells and were associated with elevated glucose uptake^27^. Interestingly, the high-yield MDCK clone C113 also demonstrates high glucose consumption, supporting the link between metabolic activity and virus yield. However, C113 displays a comparatively low proliferation rate, in contrast to MDCK-2B6, suggesting that high virus production is not solely dependent on rapid cell growth. These findings point to a complex interplay of factors (including susceptibility, metabolism, and growth dynamics) that influence the productivity of MDCK-derived producer cell lines.

Our results revealed that C113 had relatively higher abundances of lipids in all classes compared to C59, with the exception of cholesterol and ELs. This observation may highlight that IAV production is better promoted in cells that can offer greater resources for the life cycle of IAV^28–30^. C113 had relatively higher levels of CE and TG compared to C59; these two lipid classes are typically stored in lipid droplets (LDs)^23^ which are dynamic organelles that function in lipid and energy homeostasis^31^. This observation suggests that LD biogenesis is elevated in C113^33^. Increased levels of LDs have been observed in cells experiencing nutrient surplus; lipids that are stored during these conditions can be mobilized for energy production during starvation or for phospholipid synthesis for use in membranes^32^. C113 consumed large amounts of nutrients (high glucose consumption rate), while having a relatively lower growth rate, which may also explain the high abundance of LD-associated lipids in C113 compared to C59. LD-associated lipid metabolism was also observed to differ significantly between C59 and C113 during IAV infection. CE and TG species were depleted in C113 at 48 and 72 hpi whereas these species accumulated in C59 at these time points. Conflicting evidence regarding LD regulation during IAV infection is reported within the literature, where evidence has been presented that there is LD accumulation in MDCK cells upon IAV infection, that LD biogenesis is crucial for IAV replication^33^, and that LDs play a significant role in restricting viral replication^34,35^. Evidence has also been presented suggesting LDs are depleted upon IAV infection via increased autophagy^36^. Our results suggested that LD-associated lipid accumulation in C59 correlated with lower virus production and LD-associated lipid depletion in C113 was associated with higher virus production. However, despite the observed TG depletion, most TG species remained more abundant in C113 compared to C59 during infection (Supplementary Fig. S7 and Supplementary Table S10). These results suggest that LD-associated accumulation during infection is less important than having an elevated number of LDs available to the virus during production to ensure high viral yields. Based on these findings, the activation of sterol regulatory element binding proteins (SREBPs) which regulate lipid biosynthesis by controlling the expression of enzymes required for cholesterol, fatty acid, triacylglycerol and phospholipid synthesis^37^ may represent a functional mechanism to improve virus production. Media supplementation of lipid mixtures has been shown to improve viral performance for the human immunodeficiency virus 1 (HIV1)^38^ and the addition of cholesterol to the culture media has been shown to stabilize and restore the infectivity of IAV particles^39^. Taken together, the activation of SREBPs may be advantageous for enhancing virus production and represents an interesting hypothesis to explore in future work.

Another striking difference between the two clones is the elevated levels of ELs (EtherPC and EtherPE), in C59 compared to C113. ELs are biosynthesized through a well-characterized process, where the alkyl moiety linking step occurs inside the peroxisome^40^. ELs have unique structural membrane properties, leading to reduced fluidity and increased rigidity^41^, as well as playing roles in membrane trafficking^42^, signaling^43^, and as cellular antioxidants^44^. Gerl et al. have reported that the EtherPE lipid class is the third most abundant phospholipid in MDCK cells^8^. The increased levels of ELs in C59 may indicate increased peroxisomal activity compared to C113, or reduced ß-oxidation, where fatty acids are not oxidized and instead used for EL biosynthesis in the peroxisome^45^. It has been shown that impaired ß-oxidation caused by pharmacological and genetic interference in A549 cells impairs IAV production^10^, which could be a potential explanation for why C59 has lower virus yields than C113. Another study, however, reported that IAV production was linked with decreased ß-oxidation and increased EL biosynthesis^45^; taken together, this literature highlights the complexity of lipid dynamics under IAV infection. In K562 cells, ELs were also identified as a robust way to modulate mitochondrial ROS as knocking down EL biosynthetic genes resulted in lower levels of mitochondrial superoxide^46^. High viral-producing clonal MDCK H1 cells were shown to regulate ROS species better than other clones^28^, demonstrating a potential correlation between elevated EL biosynthesis and lower viral yields for IAV production. These results suggest that inhibiting alkylglycerol phosphate synthase (AGPS) to reduce EL biosynthesis using small molecules^47^ or genetic alterations^48^ may represent an opportunity to enhance IAV production.

The biomolecular complexity of IAV infection in C59 and C113 cells was also observed through DGs. DG species, which are intermediates in GPL biosynthesis and act as lipid second messengers, were consistently elevated in infected C59 relative to infected C113 across all timepoints. This increase correlated with a widespread phospholipid depletion, a pattern also observed in H292 and A549 cells during IAV infection, where it was hypothesized that the rise in DG resulted from enhanced degradation of GPLs induced by viral infection^15,49^. LPC species also exhibited changes during infection, highlighting another lipid class potentially involved in modulating IAV production. LPC species were depleted throughout infection in both C59 and C113 with similar observations reported in IAV-infected H292 and A549 cells^15^. LPC species can be biosynthesized by deacylation of PC species via phospholipase A2 (PLA2) and can be reacylated by lysophosphatidylcholine acyltransferase (LPCAT) to form PC species^50^, establishing an equilibrium between these two lipid classes, which was observed at 24 and 48 hpi in C113. The PC/LPC equilibrium dynamics that we observed may represent increased activity in LPCAT and may highlight its role as a contributor to high viral yield. LPC species can inhibit membrane fusion during viral entry due to its positive curvature effect on membranes^51^. LPC species have also been described to inhibit IAV fusion upon host entry by binding to fusion peptides thereby preventing their interaction with the target membrane^52,53^, further reinforcing the hypothesis that LPCAT activation may promote more efficient virus production by reducing LPC levels.

While changes in cellular lipid profiles reflect the host cell response to infection, the lipid composition of the viral envelope directly influences the transduction functionality and quality of the viral particles themselves^11,12^. For example, modulation of the biosynthetic pathway of SMs has been shown to directly impact the transduction potential of progeny virions^10,54–59^. In C113, SM levels decreased at 48 hpi, correlating with the highest infectious and total titers, suggesting that SM species are possibly recruited by the virus during high infectious particle production phases. Despite C59 achieving its highest total titer at 48 and 72 hpi, these timepoints correlated with low infectious titers, implying the production of defective particles. Similarly at 72 hpi in C113, the total titer remained similar to 48 hpi; however, the infectious titer nearly decreased by 30%, signifying an increase in defective, incomplete, or degraded particles. This decrease in infectious particles correlated with an increase in SM species in both cell clones, suggesting that IAV could be budding without acquiring sufficient SM species for efficient transduction in its viral lipid envelope. Insufficient SM levels in the host and the IAV particles have been demonstrated to impact the ability for IAV to infect without impacting HA levels by impairing attachment and internalization^60^, strongly suggesting the involvement of lipids, specifically SM species for successful infection and replication. Broader analysis of the viral envelope revealed a consistent enrichment of lipid raft-associated lipids (cholesterol, SM, and GSLs) across both IAV C59 and IAV C113, further emphasizing the structural importance of specific lipid domains in IAV production. This observation also suggests that the composition of lipid rafts is similar in both C59 and C113 as IAV procures its lipid envelope from lipid rafts^8,9^.

Despite these shared lipid raft features, distinct differences in phospholipid composition, particularly the enrichment of PE species in IAV C113 may contribute to variations in membrane dynamics and viral infectivity between IAV C59 and IAV C113. PE species are known for their intrinsic negative curvature, which promote membrane fusion by stabilizing the non-lamellar intermediate structures in the fusion process due to its cone shape^61^. Consequently, they play a role in viral fusion and therefore infectivity^62,63^. IAV grown in chicken eggs has been shown to have a lipid envelope highly abundant in PE, and since it has such large influence on membrane properties, it is likely that the variations in PE levels impact virus production during infection^11^. Human cytomegalovirus has been shown to be enriched in PE species and depleted in PS species compared to the host cell membrane, mimicking synaptic vesicles promoting budding and release^64^. Since IAV C113 is enriched in PE species and depleted in PS species compared to IAV C59, this specific viral membrane composition signature may serve as an indicator of high yield IAV particles. Additionally, the subtle differences in lipid composition of the viral lipid envelope between IAV C59 and IAV C113 could influence the stability of IAV particles and the immunogenicity of influenza vaccines. Evidence demonstrates that the stability of lipid nanoparticles (LNPs) used for mRNA vaccines is determined by a multitude of factors, including their lipid composition^65,66^. Differences in LNP composition for adjuvant formulation in quadrivalent influenza vaccinated mice was found to influence immune responses subsequent to infection and was determined to be an important consideration for vaccine safety and efficacy^67^. Accordingly, it would be of interest to investigate whether differences in the lipid envelopes of IAV produced from different cell clones similarly affect influenza vaccine stability and immunogenicity.

At present, establishing mechanistic linkages between lipidomics and viral yield remains a challenge, with many studies reporting different conclusions and employing different cell lines and virus strains. Multiple lipidomics studies have complemented their observations with transcriptomic and/or proteomic data to attempt to determine a causal relationship between a certain lipidomic effect and biological outcome. In this study, the data presented contains limitations and is largely correlative in nature. While the lipid alterations are statistically consistent, their mechanistic role in viral productivity remains unconfirmed and speculative. Moreover, the low number of biological replicates represents a statistical limitation during the data analysis, increasing the likelihood of false negatives and the impact of possible outliers on the dataset. However, this study serves as a strong starting point for future, larger studies that will aim to establish mechanistic insights and improve viral yields leveraging the information generated in this work. Experimental studies to determine causal effects such as AGPS inhibition and SREBP overexpression as mentioned previously and their impact on viral yields and lipid profiles would be beneficial to establish a mechanism of action and pinpoint specific lipid modulations responsible for high yields.

Despite the correlative nature of this work, the results reported here indicate that lipid dynamics could contribute in complex ways to the infectivity of IAV particles. SM levels appear tightly correlated to the production of infectious viral particles, with insufficient incorporation potentially contributing to the formation of defective particles. The conserved enrichment of lipid raft-associated lipids such as SM, GSLs, and cholesterol in both IAV C59 and IAV C113 underscores the importance of these domains in viral assembly. These observations support the possible integration of targeted lipidomics methods in biomanufacturing pipelines to monitor the quantity of SM species (e.g.: SM d34:1, SM d42:2, SM d42:1) and lipid raft associated lipids. Although such analyses have not yet been widely applied in the context of biomanufacturing, the results herein suggest they could be valuable for research and development, with potential to optimize viral yield and product consistency. Additionally, this approach presents a promising new avenue for quality assurance by providing lipid-based metrics in the production of IAV. Moreover, clone-specific subtle differences in phospholipid composition, particularly the enrichment of PE and depletion of PS in C113, suggest that finding methods to control the host lipidome may lead to novel strategies to enhance viral yield.

To the best of our knowledge, this work demonstrated for the first time that monoclonal MDCK cell lines have unique lipid profiles and dynamics upon IAV infection. Furthermore, despite differences in host lipidomes and lipid dynamics following infection, the lipid envelopes of the purified IAVs from the two monoclonal MDCK cell lines were quite similar. This work identified key lipid compositions and dynamics that can be found in the noninfected and infected host cells, and the final viral product. The lipid composition and dynamics signatures reported here provide valuable insight into virus-host interactions and open the path to the development of refined biomanufacturing strategies and quality assurance metrics for the production of IAV.

## Materials and methods

### Cell culturing, infection and sample preparation

Monoclonal suspension MDCK cell lines C59 and C113^7^ (Sartorius, Germany) were cultivated in 4Cell^®^ MDXK CD medium (Sartorius, Germany) supplemented with 8 mM GlutaMAX (Gibco, Thermo Scientific, USA) in non-baffled shake flasks with 30, 70 or 120 mL working volume. Cells were passaged 2–3 times a week using an inoculation density of 5 × 10^5^ cells/mL and were maintained at 37 °C and 5% CO_2_ atmosphere with a shaking frequency of 175 rpm (19 mm throw). VCC and diameter were measured with a Vi-CELL XR automated cell counter (Beckman Coulter, USA).

For cellular lipid analysis, cells were centrifuged at 300 × g for 5 min at room temperature and seeded at 2 × 10^6^ cells/mL in fresh medium containing 3% TrypLE Select Enzyme (10X) (Gibco, Thermo Scientific, USA). Cells were infected at a multiplicity of infection of 10^− 3^ using influenza A/Puerto Rico/8/1934 (H1N1) seed virus that was propagated in adherent MDCK cells. After IAV infection, once per day, a total cell number of 2 × 10^6^ cells was collected via a centrifugation step at 3,000 × g for 5 min. Supernatant was aliquoted for titration and metabolite analysis; pellets were washed twice with PBS, snap-frozen using dry ice and stored at -80 °C until lipidomics analysis. Experiments were carried out in biological duplicates with uninfected cells serving as controls. Experiments were limited to biological duplicates (*n* = 2) due to the expensive nature of the work and limited accessibility to the monoclonal cell lines.

To investigate the lipid profile of IAV, cells were infected as described above and the virus was harvested after 48 h from the supernatant by stepwise centrifugation. Cells and debris were removed at 300 × *g* for 10 min and 1,200 × *g* for 20 min. The supernatant was cleared using a 0.45 µM Whatman Uniflo PES syringe filter and was subjected to ultracentrifugation at 114,000 × *g* and 4 °C for 90 min. Pelleted IAV was reconstituted in 1 mL PBS, thoroughly mixed using a 3 cc syringe with decreasing needle taper (18G, 25G, 30G) and overlaid on a 10–30% OptiPrep (Sigma-Aldrich, USA) gradient. Following ultracentrifugation at 186,700 × *g* and 4 °C for 120 min, the virus band was collected and stored at -80 °C until lipidomic analysis.

### Metabolite analysis

Concentrations of glucose and lactate were determined in single measurements with the Cedex Bio Analyzer (Roche, Switzerland). The measurement range was previously validated for each metabolite and samples were diluted accordingly when out of range.

### Virus and quantification

To quantify the total virus load based on the hemagglutinin (HA) surface protein, an ELISA specific to influenza A/Puerto Rico/8/1934 HA (SinoBiological, China) was employed following the manufacturer’s instructions. Briefly, samples and standards were diluted appropriately and added to the pre-coated and washed ELISA plate, followed by incubation for 2 h. Plates were then washed again before adding the antibodies and incubating for another hour. After a final wash, substrate solution was added, and the reaction was stopped after 20 min. Absorbance was measured at 450 nm, and HA concentrations were determined using linear regression based on the standard curve, expressed as pg/mL.

A plaque assay was used to determine the infectious virus titer. Confluent monolayers of adherent MDCK cells were infected with 100 µL of serial 10-fold dilutions of each sample. After 1 h of incubation, the inoculum was removed, and cells were overlaid with a pre-warmed solution containing 2x MEM and 1.3% agar (1:1) with 0.01% TPCK trypsin. Plates were incubated for 2 days at 37 °C in a 5% CO₂ atmosphere. Subsequently, 3 mL of Carnoy’s Fixative (methanol and glacial acetic acid, 3:1) was added to each well, and plates were fixed for 2 h. After removing the overlay, plates were stained with Coomassie Blue, and plaques were counted in at least two dilutions to calculate PFU per mL.

### Lipid extraction

Additional details on the lipid extraction procedure are included in the Supplementary Material. Lipids were extracted using a modified Bligh-Dyer method^68^ as described in Roberts et al., 2024^21^.

### Data acquisition

Additional data acquisition details are included in the Supplementary Material. All data was acquired using an Agilent 6546 QToF coupled to an Agilent 1260 HPLC as previously reported^21^. Samples were sorted randomly and first injected with the QToF acquiring MS-level only scans, each sample was analyzed in both positive and negative polarity via sequential injections. Technical triplicates were recorded for all samples in each polarity. A quality assurance interval consisting of a blank sample consisting of 100% methanol and a fetal bovine serum lipid extract sample was injected in both polarities after every 18 samples to evaluate sample carry-over and instrument performance. Instrument performance parameters (mass accuracy and retention time drift) are included in Supplementary Figure S8. Replicates for each sample were then pooled together and injected twice in positive and negative polarity using a data-dependent MS/MS acquisition with the iterative injections feature activated for lipid identification^69^.

### Data analysis

Additional data analysis details are included in the Supplementary Material. Lipids were identified in pooled samples using Agilent Lipid Annotator^70^. Cholesterol was manually identified as the [M-H_2_O + H]^+^ ion in positive polarity^71,72^. Identification was confirmed by retention time and exact mass using cholesterol *d*7 (Supplementary Fig. S9). A list of abbreviations for all lipid classes detected in the analysis is provided in Supplementary Table S11. MS data files were converted to .mzML format using msConvert^73,74^ and imported into MZmine 4.1.0^75^ for feature detection and feature list alignment. Feature lists were exported into R (R-4.0.3) for normalization and imputation. Log_2_ fold changes were calculated by averaging the replicates (*n* = 6 for cell samples, *n* = 3 for virus samples) in each group and calculating the difference between the “treatment” and “control” groups. All *p* values were calculated using Student’s t-test (2-tailed, unpaired with unequal variance) with FDR-correction using all replicates (*n* = 6 for cell samples, *n* = 3 for virus samples). The double bond index was determined by calculating the weighted average of the number of double bonds on each lipid per class. The normalized peak area was used as the weights. The same calculation was performed to determine the weighted average chain length.

## Supplementary Information

Below is the link to the electronic supplementary material.

Supplementary Material 1

Supplementary Material 2

## Data Availability

Raw LC-MS data files are available on the MassIVE repository (MSV000097713).
