# Supplementary material for: Lipidomic profiling of influenza A virus production in MDCK cells towards targeted clone selection
Source: Sci Rep. 2026 Jan 7;16:882. doi: 10.1038/s41598-025-33499-1 (PMC12783198; doi:10.1038/s41598-025-33499-1)
Supplement: Supplementary file 2 — Supplementary Material 2 [file 41598_2025_33499_MOESM2_ESM.docx]

**Supplementary Information**

**Lipidomic profiling of influenza A virus production in MDCK cells towards targeted clone selection**

^¶^Jocelyn A. Menard ^1^, ^¶^Tilia Zinnecker ^2^, Elena Godbout ^3^, Joshua A. Roberts ^1^, Rozanne Arulanandam ^3^, Andrew Chen^3^, Anne Landry^3^, Christopher N. Boddy ^4^, Udo Reichl ^2,5^, Jean-Simon Diallo ^3,6^, ^*^Yvonne Genzel ^2^, ^*^Jeffrey C. Smith ^1,7,8^

^1^ Department of Chemistry, Carleton University, Ottawa, ON, Canada

^2^ Max Planck Institute for Dynamics of Complex Technical Systems, Magdeburg, Germany

^3^ Centre for Cancer Therapeutics, Ottawa Hospital Research Institute, Ottawa, ON, Canada

^4^ Department of Chemistry and Biomolecular Sciences, University of Ottawa, Ottawa, ON, Canada

^5^ Chair of Bioprocess Engineering, Otto von Guericke University, Magdeburg, Germany

^6^ Department of Biochemistry, Microbiology and Immunology, University of Ottawa, Ottawa, ON, Canada

^7^ Institute of Biochemistry, Carleton University, Ottawa, ON, Canada

^8^ Carleton Mass Spectrometry Centre, Carleton University, Ottawa, ON, Canada

* Corresponding authors

E-mail: JeffCSmith@CUNET.CARLETON.CA (J. C. S.), [genzel@mpi-magdeburg.mpg.de](mailto:genzel@mpi-magdeburg.mpg.de) (Y. G.)

^¶^These authors contributed equally to this work

**Table of Contents**

| **Title** | **Page Number** | **Figure Caption** |
| --- | --- | --- |
| Supplementary Methods | S4 | Lipid extraction. |
| Supplementary Methods | S4 | Data acquisition parameters. |
| Supplementary Methods | S5 | Data analysis parameters. |
| Supplementary Fig S1 | S7 | **Cell growth parameters for C59 and C113 cell clones under control and infected conditions.** **(a)** VCC and viability of C59. **(b)** VCC and viability of C113. **(c)** Diameter measurements of C59. **(d)** Diameter measurements of C113. |
| Supplementary Fig S2 | S8 | **Relative intensity comparison of lipid classes of C59 and C113 cell clones (a)** Major lipid classes. **(b)** Lipid classes predominantly found in the plasma membrane. All *p* values were calculated using Student's t-test (2-tailed, unpaired with unequal variance) with FDR-correction (n=6, two biological replicates with three technical replicates). Shown in red under the figure titles: NS, *p* > 0.05, *, *p* ≤ 0.05, **, *p* ≤ 0.01, ***, *p* ≤ 0.001. Error bars indicate the standard deviation between replicates. |
| Supplementary Fig S3 | S9 | **Metabolite analysis of C59 and C113 cell clones after infection (n=2).** **(a)** Glucose consumption rate. **(b)** Glucose concentration. **(c)** Lactate concentration. |
| Supplementary Fig S4 | S10 | **The log_2_ relative intensity of ceramide and hexosylceramide lipid classes in control and infected samples of C59 and C113 cell clones at each timepoint.** Statistical comparison between control and infected samples are represented above the x axis with each shape representing a timepoint and the color representing a positive or negative log_2_FC. All *p* values were calculated using Student's t-test (2-tailed, unpaired with unequal variance) with FDR-correction (n=6, two biological replicates with three technical replicates). |
| Supplementary Fig S5 | S10 | **Bar plots representing the intensity ratio between phosphatidylcholine species and lipids commonly found in lipid rafts of control, infected, and virus samples.** **(a)** glycosphingolipids, **(b)** sphingomyelin, **(c)** cholesterol, **(d)** ceramides. Error bars indicate the standard deviation between replicates (n=6 for cell samples, n=3 for virus samples). |
| Supplementary Fig S6 | S11 | **Fatty acid chain analysis of IAV C59 and IAV C113. (a)** Box plots representing the weighted average total chain length of IAV C59 and IAV C113 for each class. **(b)** Box plots representing the double bond index of IAV C59 and IAV C113 for each class. All *p* values were calculated using Student's t-test (2-tailed, unpaired with unequal variance) with FDR-correction (n=3, one biological replicates with three technical replicates). Shown in red under the figure titles: NS, *p* > 0.05, *, *p* ≤ 0.05, **, *p* ≤ 0.01, ***, *p* ≤ 0.001. |
| Supplementary Fig S7 | S12 | **Bubble plots representing the fold change of TG species after infection of C59 and C113 cell clones.** Positive values indicate a greater abundance for C113, negative values indicate a greater abundance for C59. Lipids with significant log_2_FC (FDR-adjusted *p* < 0.05) are bolded. Lipids on x axis are sorted based on alphabetical order. The size of the symbols is proportional to the -log_10_(FDR-adjusted *p* value). **(a)** 24 hpi **(b)** 48 hpi **(c)** 72 hpi. All *p* values were calculated using Student's t-test (2-tailed, unpaired with unequal variance) with FDR-correction (n=6, two biological replicates with three technical replicates). |
| Supplementary Fig S8 | S13 | **LC-MS/MS performance across all analyses. (a)** Mass accuracy of four internal standards in positive polarity. **(b)** Mass accuracy of three internal standards in negative polarity. Retention time range of four internal standards **(c)** CE 17:0, **(d)** LPC 13:0, **(e)** LPE 13:0, **(f)** PC 19:0-19:0. Error bars indicate the standard deviation between replicates (n=12 for **a-c**, n=24 for **d-f**). |
| Supplementary Fig S9 | S13 | **Cholesterol identity confirmation using cholesterol-*d*7 and monitoring the retention time of the [M-H_2_O+H]^+^ ion.** |

**Supplementary Methods**

**Lipid extraction**: Lipids were extracted using a modified Bligh-Dyer method [1]. Briefly, the sample was transferred to conical 10 mL glass centrifuge tubes (Kimble, USA). 1 mL of water with 0.1 M sodium acetate and 2 mL of methanol with 2% acetic acid (v/v) were added to each tube and then homogenized by hand and bath sonicated for 5 min. Internal standards (CE 17:0, LPC 13:0, LPE 13:0, and PC 19:0-19:0 (Avanti Research, USA)) were spiked in to reach a final concentration of 2 µM, except for CE 17:0, where the final concentration was 10 µM. 1.5 mL chloroform was then added to each tube and shaken for 2 min, then centrifuged (528 × *g*, 2 min). The chloroform layer was carefully removed using a Pasteur pipette and transferred to a new 10 mL glass centrifuge tube. The water/methanol layer was then extracted two more times with 1 mL of chloroform, and each chloroform layer was combined with the previous extractions for a total of 3.5 mL per sample. The chloroform was then evaporated under a stream of nitrogen gas and dissolved in 250 µL of 1:1 methanol:ethanol for cell samples and 70 µL for virus samples, incubated at 30°C for 10 min, centrifuged (528 × g, 2 min), and transferred to an amber HPLC vial containing a glass insert with a PTFE cap.

**Data acquisition**: All data were acquired using an Agilent 6546 QToF sequentially operating in both positive and negative polarities coupled to an Agilent 1260 HPLC system using MassHunter Acquisition Software (version 10.0). An Agilent Poroshell 120 EC-C18 (2.7 µm, 2.1x100 mm) column was used for all analyses. The HPLC column compartment was heated to 45 ^o^C, injection volumes of 5 and 10 µL were used for positive and negative mode, respectively, and the flow rate was set to 400 µL/min. The following MS source parameters were used for all analyses: gas temp 200 ^o^C , drying gas 10 L/min, nebulizer 50 psi, sheath gas temp 300 ^o^C, sheath gas flow 12 L/min, VCap 3500 V, fragmentor 150 V, skimmer 75 V, Oct 1 RF Vpp 750 V, mass range of m/z 40-1700 and an acquisition rate of 3 spectra/s. Mobile phase solvent A contained water:methanol (1:1 v/v) (LiChrosolv, Sigma-Aldrich, USA) with 10 mM ammonium formate (LiChropur, Sigma-Aldrich, USA), and solvent B was methanol:isopropanol (1:3 v/v) (LiChrosolv, Sigma-Aldrich, USA) with 10 mM ammonium formate. The following HPLC gradient was used for analyses in positive polarity: 0 min 20% B, 0.35 min 20% B, 0.4 min 32% B, 9.6 min 44% B, 9.7 min 65% B, 11.5 min 65% B, 26.8 min 82%, 27.3 min 87% B, 37 min 96% B, 37.1 min 100% B, 44 min 100% B. Injections in negative ion mode used a modified gradient program: 0 min 20% B, 0.35 min 20% B, 0.4 min 32% B, 9.6 min 44% B, 9.7 min 65% B, 11.5 min 65% B, 30.0 min 86% B, 30.1 min 100% B, 39 min 100% B. The HPLC was operated at 20% B for 5 minutes following each injection to re-equilibrate the column. Samples were sorted randomly and first injected with the QToF acquiring MS-level only scans, each sample was analyzed in both positive and negative polarity with sequential injections. Technical triplicates were recorded for all samples in each polarity. A quality assurance interval consisting of a blank sample consisting of 100% methanol and a fetal bovine serum lipid extract sample was injected in both polarities after every 18 samples to determine sample carry-over and instrument performance. The MS was calibrated in each polarity prior to every batch and after 24 hours of continuous analysis. Once triplicates were recorded for each sample in both polarities at MS-level only, replicates for each sample were pooled together and injected using a data-dependent MS/MS acquisition method with a quadrupole isolation width of 1.3 m/z and the following settings: 10 precursors/cycle, absolute threshold 5000 counts, active exclusion enabled after 1 spectra and released after 0.15 min, abundance dependent accumulation of 25000 counts/spectrum, purity stringency of 70% and a purity cut-off of 0%. Each sample was injected twice in positive and negative polarity using the same data-dependent MS/MS with the iterative injections feature activated [2]: excluding features ± 20 ppm and ± 0.15 min RT from the previous injection.

**Data analysis**: Lipids were identified in pooled samples using Agilent Lipid Annotator. All annotations were exported as a .csv file and reconfigured to a format suitable to serve as the input for the “Targeted Feature Extraction” module in MZmine. Cholesterol was manually identified as the dehydrated fragment ion, *m/z* 369.3521 [M-H_2_O+H]^+^ ion in positive polarity [3,4]. The identification of cholesterol was confirmed by retention time and exact mass using cholesterol *d*7 (Supplementary Fig. S9). A list of abbreviations for all lipid classes detected in the analysis is provided in Supplementary Table S11. MS-level only data files were converted to .mzML format using msConvert [5,6] and imported into MZmine 4.1.0 [7]. All files were processed with the mass detection module using the centroid detector and 5E3 noise levels. The targeted feature extraction module was then used with the following settings: 25% intensity tolerance, m/z tolerance of 0 *m/z* or 10 ppm, and an RT tolerance of ± 0.10 min (absolute). RTs on feature lists were calibrated using the following settings: *m/z* tolerances 0 or 10 ppm, RT tolerance ± 0.3 min (absolute) and minimum standard intensity of 1E4. Features were then aligned using the “Join aligner” module with the following settings: *m/z* tolerance 0 *m/z* or 10 ppm, weight for *m/z* 50, RT tolerance ± 0.30 min, weight for RT 50 and were required to have the same identity. Internal standards were monitored in each sample to assess the MZmine module settings for mass and RT accuracy during data preprocessing (Supplementary Fig. S8). The steps were performed on files for each polarity separately, then peak areas were exported from MZmine and combined into the same feature list. Features were removed from the dataset following the “modified 80% rule”, where features were only kept if they were detected in 80% of samples from at least one treatment group. For host cell lipid analysis, feature lists were then imported into R (R-4.0.3), normalized using probabilistic quotient normalizing (PQN) using only structural lipid classes (Cholesterol, PC, PE, PG, PI, PS, SM) [8], and log_2_ transformed. For IAV lipid analysis, data was pre-normalized to the number of viral particles for each sample according to the plaque assay performed following Optiprep and then log_2_ transformed. IAV data was normalized using normalizeCyclicLoess from the Limma package (ver. 3.46.0) [9]. Missing values were imputed using the Quantile Regression Imputation of Left-Censored (QRILC) method impute.QRILC from the imputeLCMD package (ver. 2.1) [10]. *P* values were calculated using Microsoft Excel using 2-tailed distributions assuming unequal variance. FDR correction of *p* values was applied using p.adjust in R. Principial component analysis (PCA) was performed using the prcomp function from the [stats package (version 3.6.2)](https://www.rdocumentation.org/packages/stats/versions/3.6.2) [11]. Internal standards were not used to normalize or correct abundances for any lipid classes during data processing.


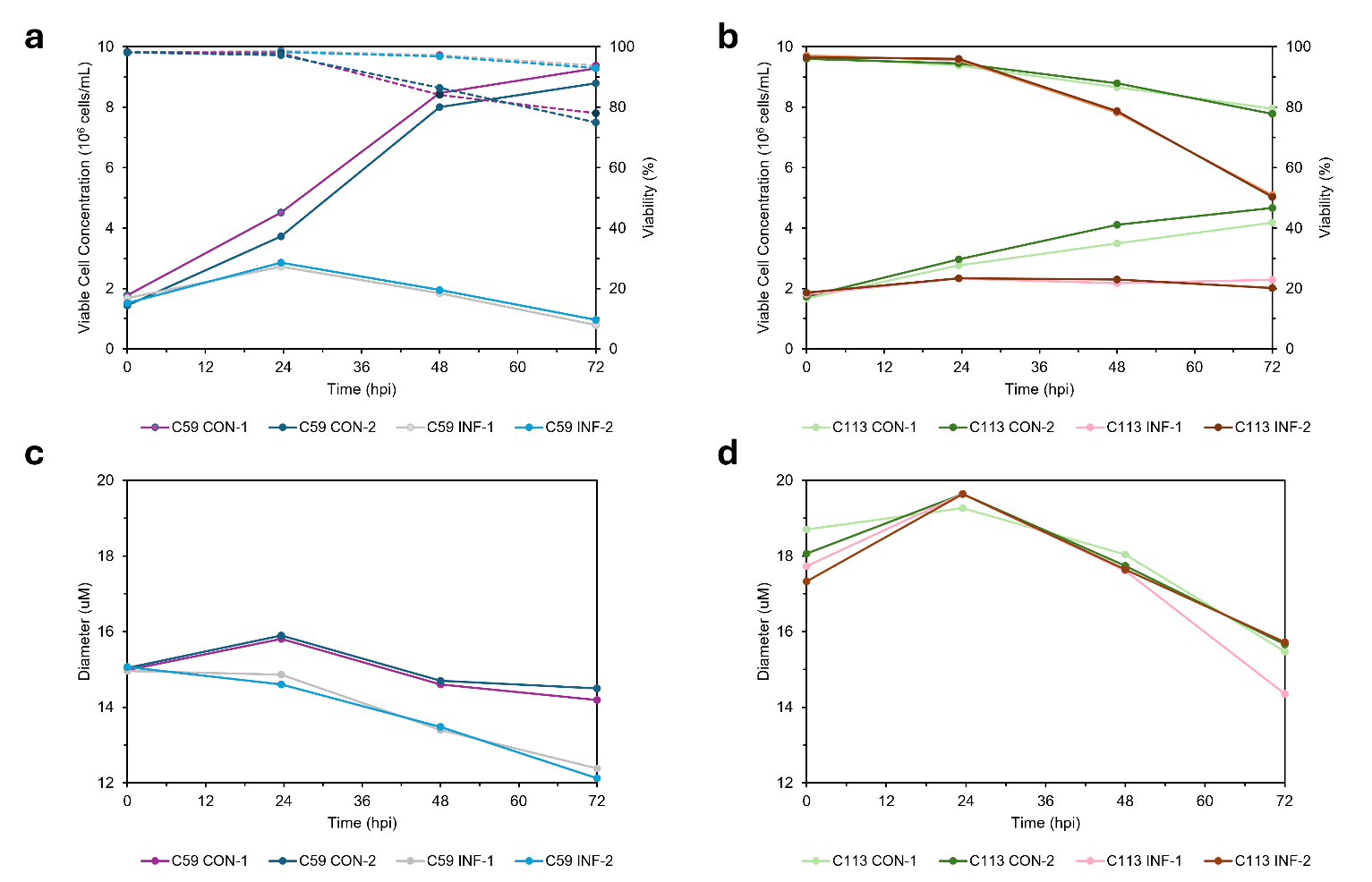


**Supplementary Fig S1.** **Cell growth parameters for C59 and C113 cell clones under control and infected conditions.** (**a)** VCC and viability of C59. **(b)** VCC and viability of C113. **(c)** Diameter measurements of C59. **(d)** Diameter measurements of C113.


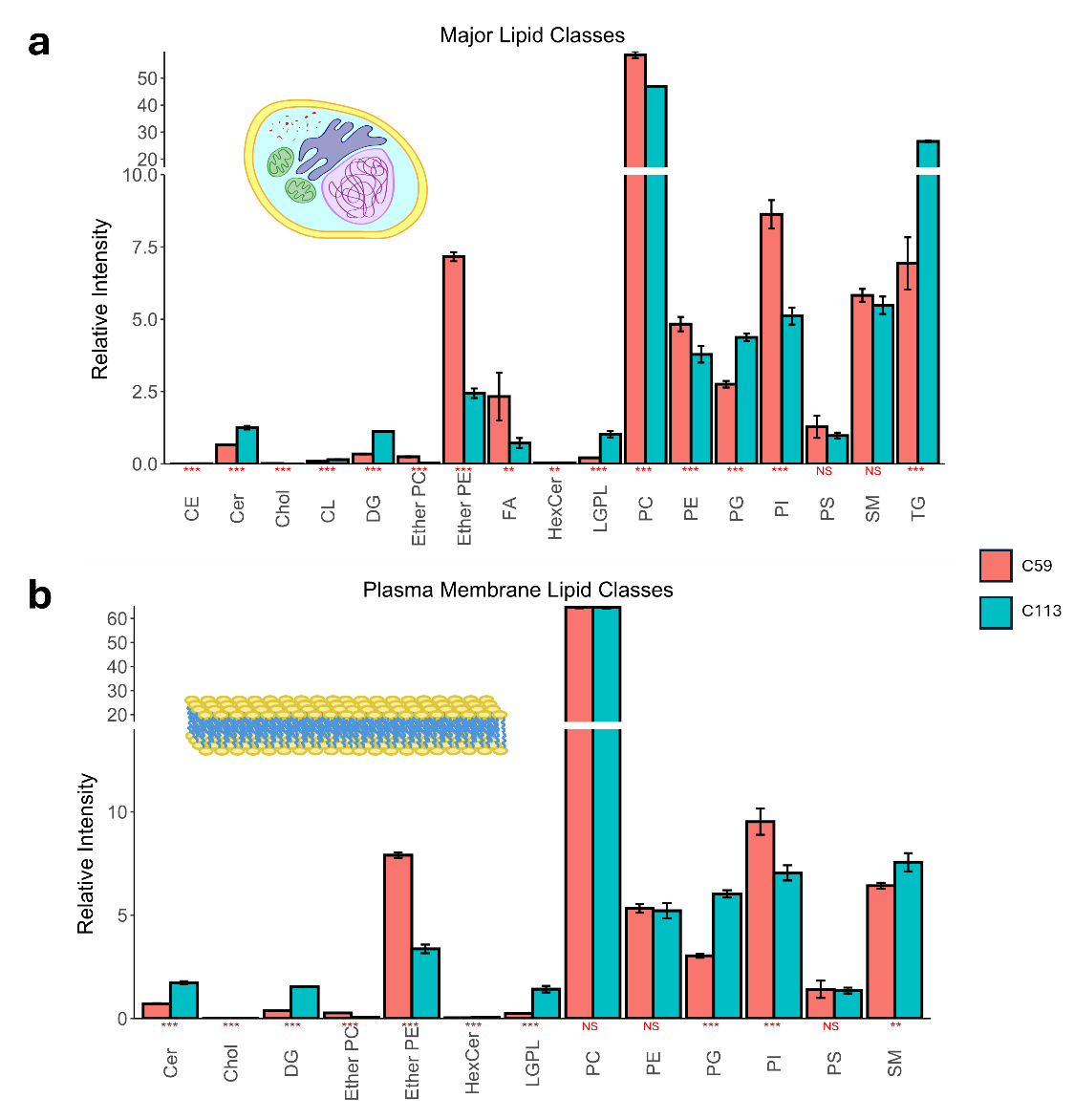


**Supplementary Fig S2. Relative intensity comparison of lipid classes of C59 and C113 cell clones (a)** Major lipid classes. **(b)** Lipid classes predominantly found in the plasma membrane. All *p* values were calculated using Student's t-test (2-tailed, unpaired with unequal variance) with FDR-correction (n=6, two biological replicates with three technical replicates). Shown in red under the figure titles: NS, *p* > 0.05, *, *p* ≤ 0.05, **, *p* ≤ 0.01, ***, *p* ≤ 0.001. Error bars indicate the standard deviation between replicates.


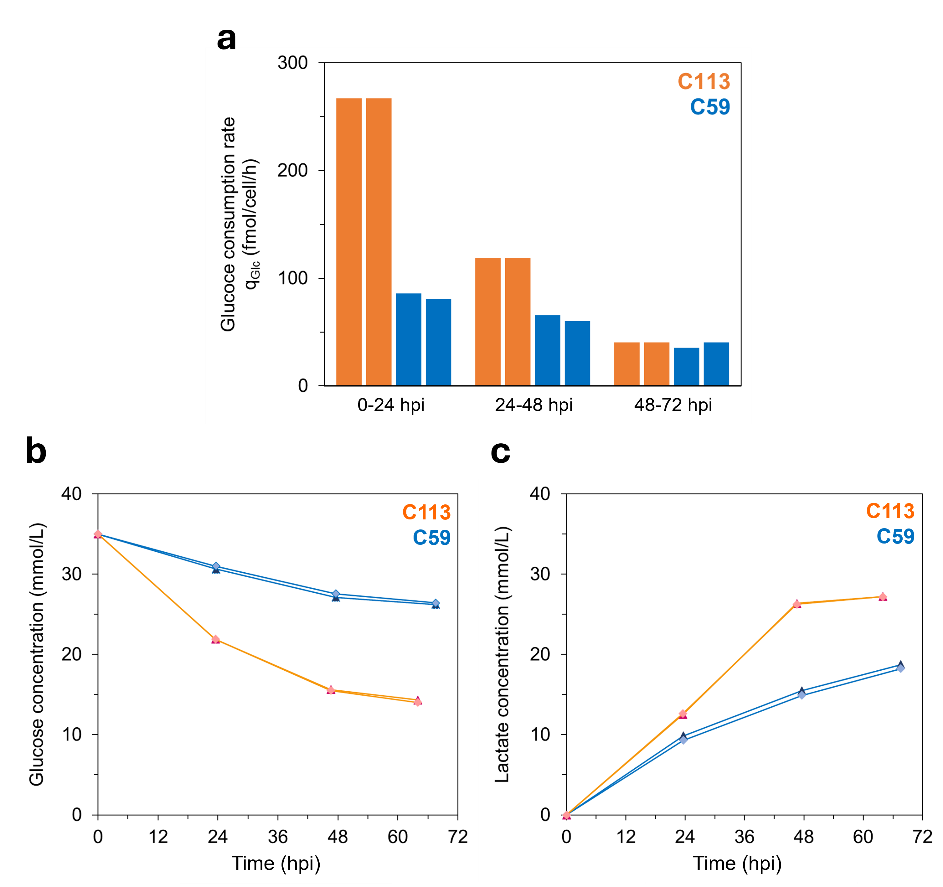


**Supplementary Fig S3. Metabolite analysis of C59 and C113 cell clones after infection (n=2).** **(a)** Glucose consumption rate. **(b)** Glucose concentration. **(c)** Lactate concentration.


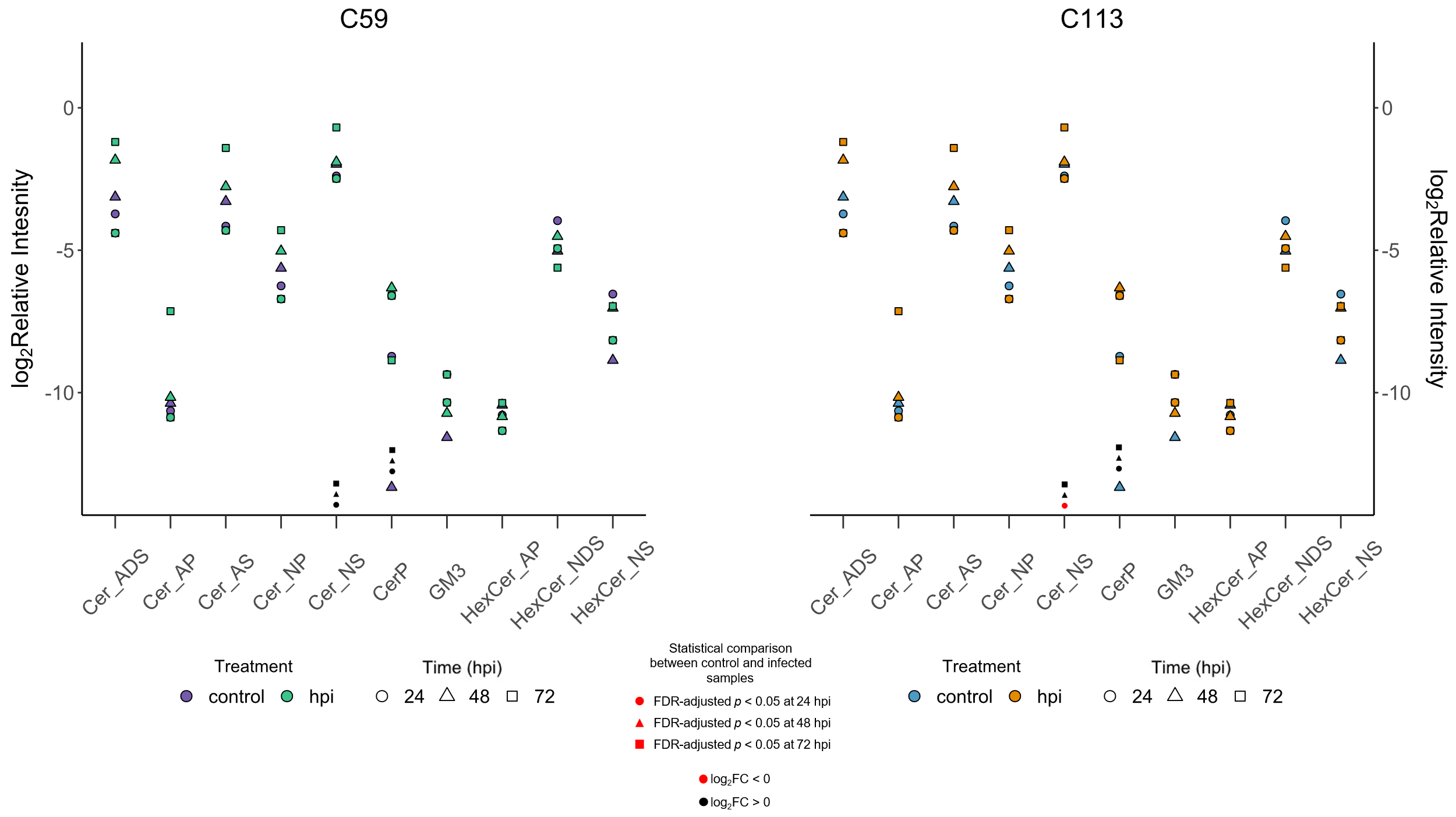


**Supplementary Fig S4. The log_2_ relative intensity of ceramide and hexosylceramide lipid classes in control and infected samples of C59 and C113 cell clones at each timepoint.** Statistical comparison between control and infected samples are represented above the x axis with each shape representing a timepoint and the color representing a positive or negative log_2_FC. All *p* values were calculated using Student's t-test (2-tailed, unpaired with unequal variance) with FDR-correction (n=6, two biological replicates with three technical replicates).


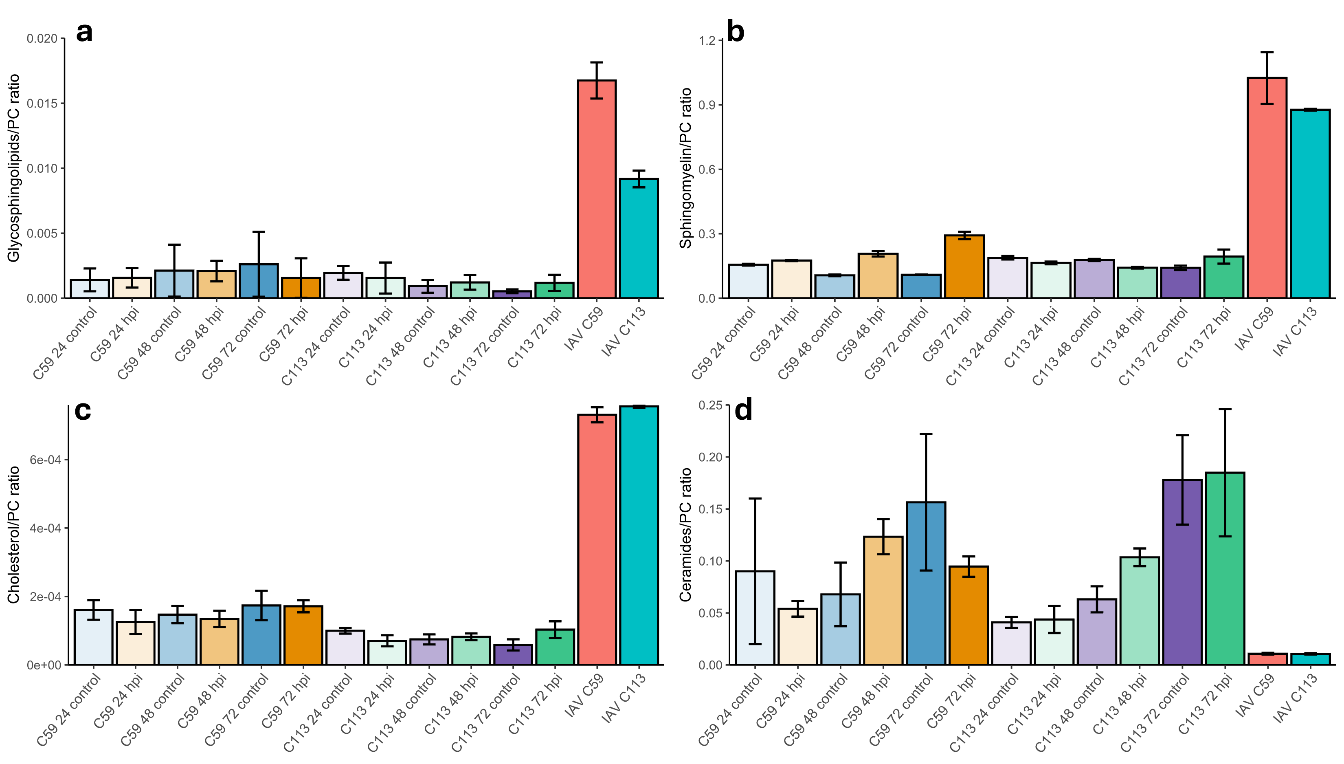


**Supplementary Fig S5. Bar plots representing the intensity ratio between phosphatidylcholine species and lipids commonly found in lipid rafts of control, infected, and virus samples.** **(a)** glycosphingolipids, **(b)** sphingomyelin, **(c)** cholesterol, **(d)** ceramides. Error bars indicate the standard deviation between replicates (n=6 for cell samples, n=3 for virus samples).


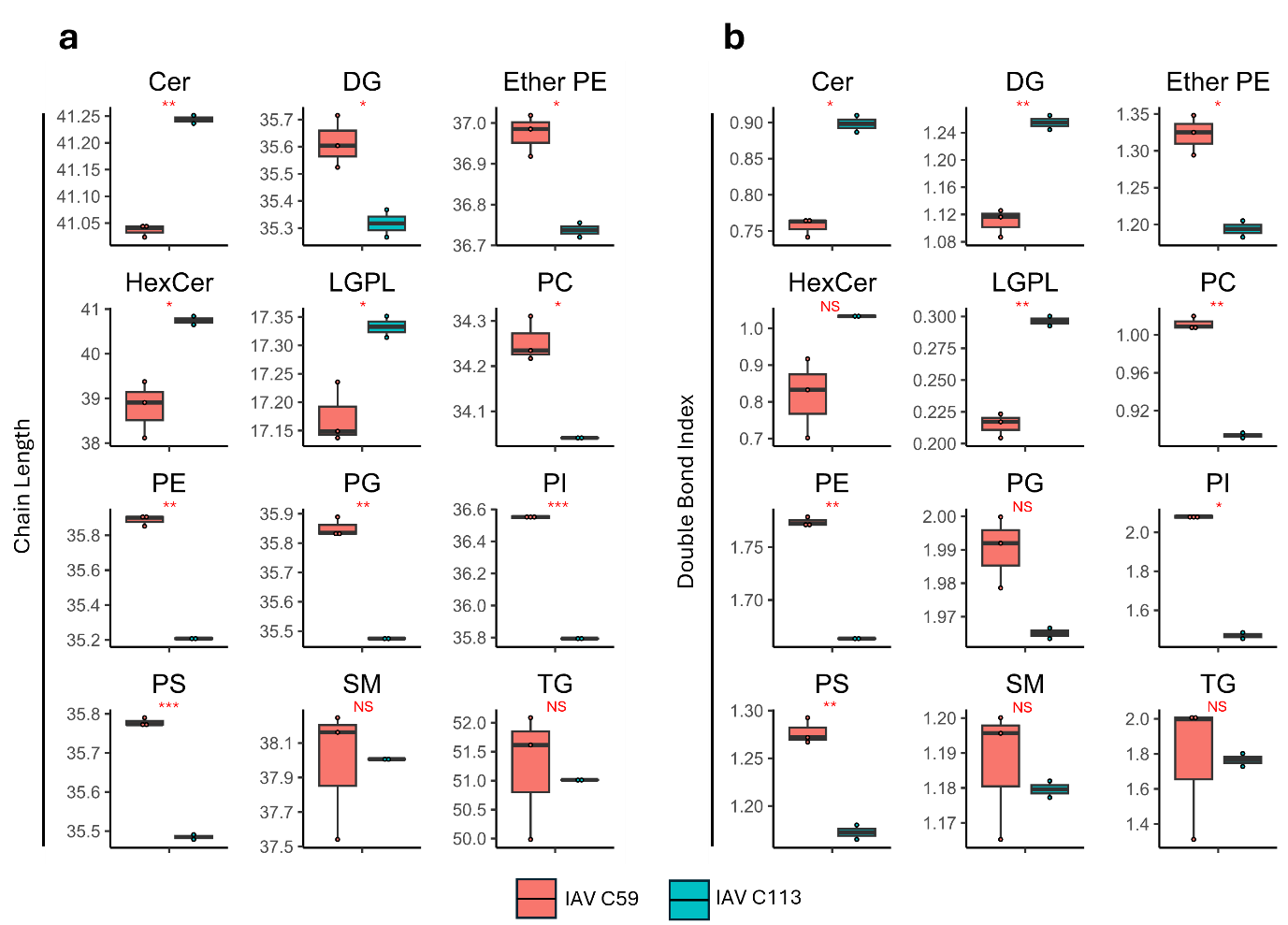


**Supplementary Fig S6. Fatty acid chain analysis of IAV C59 and IAV C113. (a)** Box plots representing the weighted average total chain length of IAV C59 and IAV C113 for each class. **(b)** Box plots representing the double bond index of IAV C59 and IAV C113 for each class. All *p* values were calculated using Student's t-test (2-tailed, unpaired with unequal variance) with FDR-correction (n=3, one biological replicates with three technical replicates).. Shown in red under the figure titles: NS, *p* > 0.05, *, *p* ≤ 0.05, **, *p* ≤ 0.01, ***, *p* ≤ 0.001.


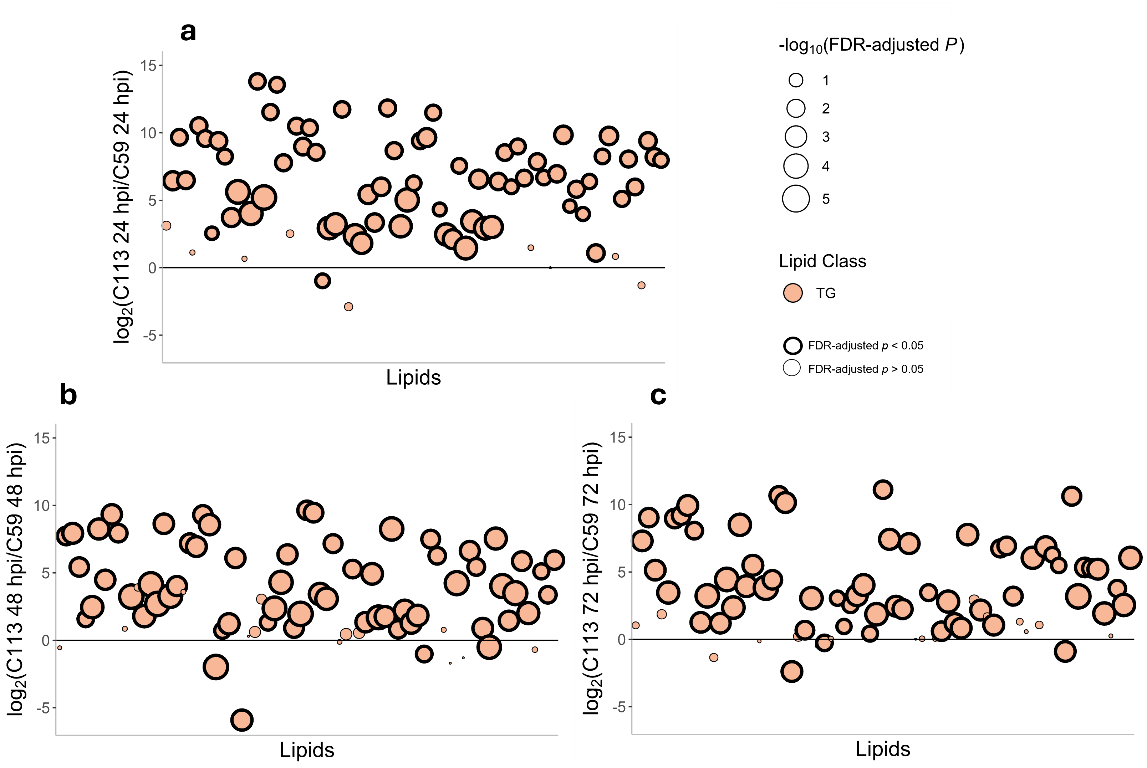


**Supplementary Fig S7.** **Bubble plots representing the fold change of TG species after infection of C59 and C113 cell clones.** Positive values indicate a greater abundance for C113, negative values indicate a greater abundance for C59. Lipids with significant log_2_FC (FDR-adjusted *p* < 0.05) are bolded. Lipids on x axis are sorted based on alphabetical order. The size of the symbols is proportional to the -log_10_(FDR-adjusted *p* value). **(a)** 24 hpi **(b)** 48 hpi **(c)** 72 hpi. All *p* values were calculated using Student's t-test (2-tailed, unpaired with unequal variance) with FDR-correction (n=6, two biological replicates with three technical replicates).


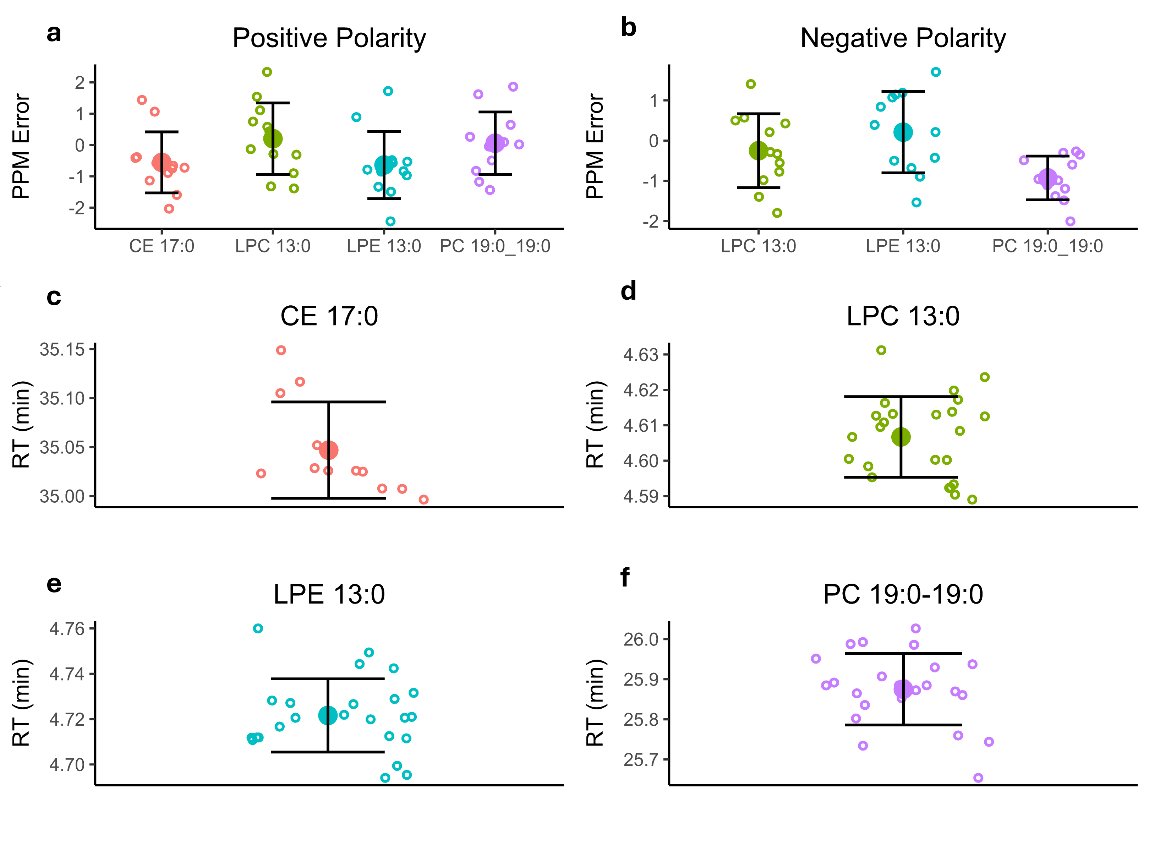


**Supplementary Fig S8. LC-MS/MS performance across all analyses. (a)** Mass accuracy of four internal standards in positive polarity. **(b)** Mass accuracy of three internal standards in negative polarity. Retention time range of four internal standards **(c)** CE 17:0, **(d)** LPC 13:0, **(e)** LPE 13:0, **(f)** PC 19:0-19:0. Error bars indicate the standard deviation between replicates (n=12 for **a-c**, n=24 for **d-f**).


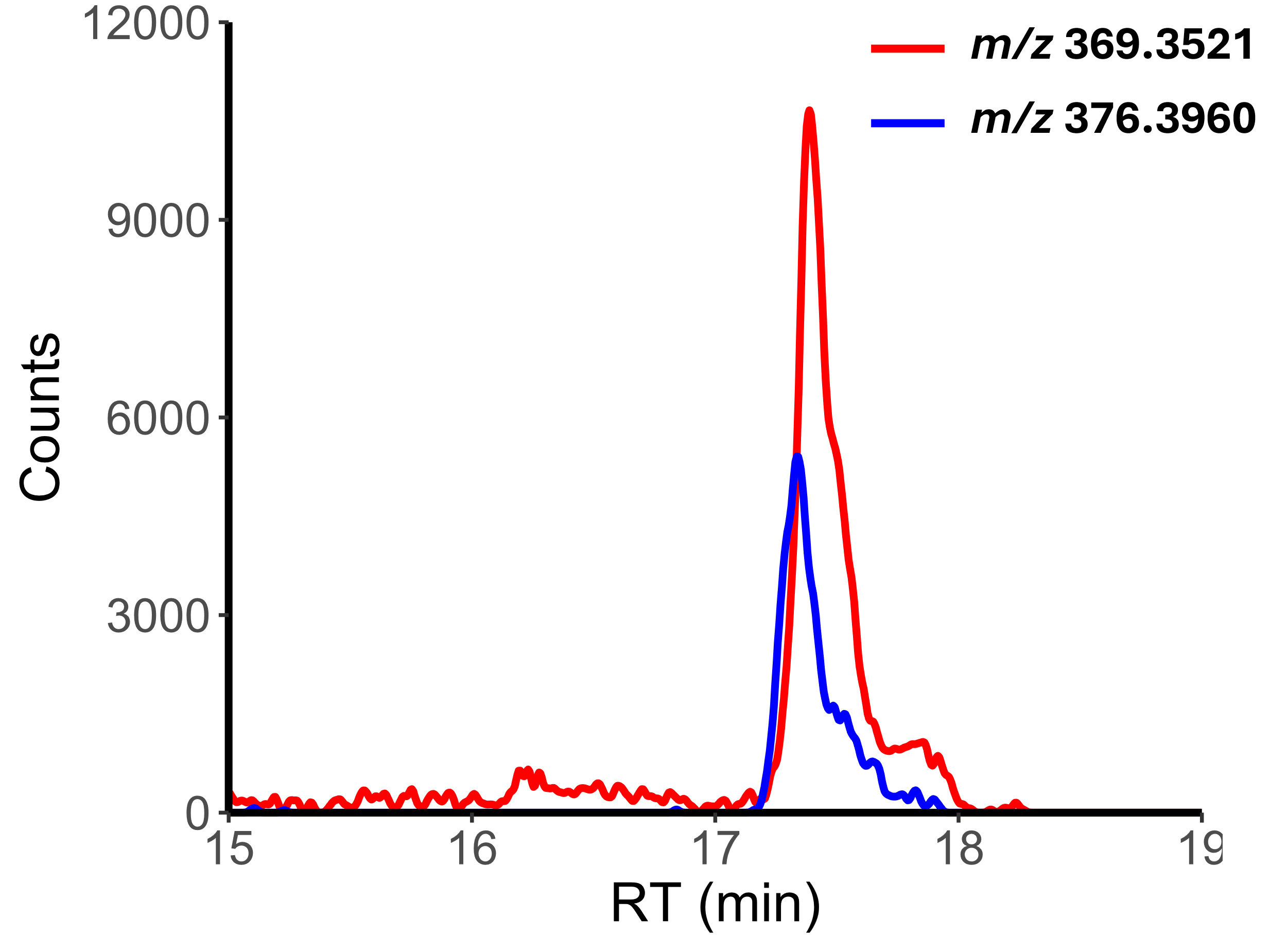


**Supplementary Fig S9. Cholesterol identity confirmation using cholesterol-*d*7 and monitoring the retention time of the [M-H_2_O+H]^+^ ion.**

# **References**

1. Bligh, E. G. & Dyer, W. J. A Rapid Method of Total Lipid Extraction and Purification. *Can J Biochem Physiol* **37**, (1959).

2. Koelmel, J. *et al.* *Improving Coverage of the Plasma Lipidome Using Iterative MS/MS Data Acquisition Combined with Lipid Annotator Software and 6546 LC/Q-TOF*. (2020).

3. Murphy, R. C. Challenges in mass spectrometry-based lipidomics of neutral lipids. *TrAC - Trends in Analytical Chemistry* **107**, 91–98 (2018).

4. Chandramouli, A. & Kamat, S. S. A Facile LC-MS Method for Profiling Cholesterol and Cholesteryl Esters in Mammalian Cells and Tissues. *Biochemistry* **63**, 2300–2309 (2024).

5. Chambers, M. C. *et al.* A cross-platform toolkit for mass spectrometry and proteomics. *Nat Biotechnol* **30**, 918–920 (2012).

6. Kessner, D., Chambers, M., Burke, R., Agus, D. & Mallick, P. ProteoWizard: Open source software for rapid proteomics tools development. *Bioinformatics* **24**, 2534–2536 (2008).

7. Schmid, R. *et al.* Integrative analysis of multimodal mass spectrometry data in MZmine 3. *Nat Biotechnol* **41**, 447–449 (2023).

8. Leegwater, H. *et al.* Normalization Strategies for Lipidome Data in Cell Line Panels. *J Chemom* **39**, (2025).

9. Ritchie, M. E. *et al.* Limma powers differential expression analyses for RNA-sequencing and microarray studies. *Nucleic Acids Res* **43**, e47 (2015).

10. Lazar, C. & Burger, T. A Collection of Methods for Left-Censored Missing Data Imputation [R package imputeLCMD version 2.1]. *CRAN: Contributed Packages* (2022).

11. Venables, W. N. & Ripley, B. D. *Modern Applied Statistics with S*. (Springer New York, New York, NY, 2002).
